# LncRNA CRLM1 inhibits apoptosis and promotes metastasis through transcriptional regulation cooperated with hnRNPK in colorectal cancer

**DOI:** 10.1186/s13578-022-00849-9

**Published:** 2022-07-30

**Authors:** Zhe Wang, Jianfang Chen, Fengjun Sun, Xiang Zhao, Yan Dong, Songtao Yu, Jianjun Li, Houjie Liang

**Affiliations:** 1 Department of Oncology and Southwest Cancer Center, Southwest Hospital, Third Military Medical University (Army Medical University), Chongqing, 400038 China; 2 Department of Pharmacy, Southwest Hospital, Third Military Medical University (Army Medical University), Chongqing, 400038 China

**Keywords:** Colorectal cancer, lncRNA CRLM1, hnRNPK, Metastasis, Gene expression

## Abstract

**Background:**

Colorectal liver metastases (CRLM) continue to have a low survival rate. The number of CRLM regulators and clinical indicators remains limited. Long non-coding RNAs (lncRNAs) are a new master regulator of cell invasion and metastasis. However, the function and regulation mechanism of lncRNAs in colorectal cancer (CRC) metastasis are yet unknown.

**Methods:**

To screen and identify CRLM-related lncRNAs, public transcriptome data were used. Gain and loss of function experiments were carried out to investigate the biological activities of lncRNA CRLM1 in vitro and in vivo. RNA sequencing (RNA-seq), chromatin isolation by RNA purification (ChIRP), immunofluorescence (IF), quantitative real-time PCR (qRT-PCR), western blotting, and rescue experiments were performed to explore the molecular mechanism of CRLM1. Moreover, identified the proteins, DNAs, and RNAs that interact with CRLM1.

**Results:**

The investigation of lncRNA expression dynamics in CRLM, primary CRC, and normal tissues in this work resulted in identifying a series of lncRNAs associated with metastasis, including CRLM1. CRLM1 inhibited apoptosis of CRC cells and promoted liver metastasis in Balb/C nude mice. CRLM1 was weakly associated with the chromatin regions of genes involved in cell adhesion and DNA damage, and this association was bidirectionally correlated with CRLM1-regulated pro-metastatic gene expression. CRLM1 physically interacts with the hnRNPK protein and promotes its nuclear localization. CRLM1 effectively enhances hnRNPK promoter occupancy and co-regulates the expression of a panel of metastatic genes.

**Conclusions:**

The finding of the clinically significant lncRNA CRLM1 in promoting metastasis and regulating gene expression suggests a potential biomarker and target for CRLM therapy.

**Supplementary Information:**

The online version contains supplementary material available at 10.1186/s13578-022-00849-9.

## Background

Colorectal cancer (CRC) is one of the fatal cancers in the world and is the third leading cause of cancer death worldwide [1, 2]. Numerous studies have shown pathogenesis and molecular regulatory mechanisms, including genomic changes and key pathways implicated in tumor growth and metastasis [3, 4]. Because of recent advances in CRC early detection screenings and treatment choices, the 5-year survival rate in stage IIIC has increased to over 50%, whereas the 5-year survival rate in stage IV has declined to 12% (https://seer.cancer.gov). These findings suggest that CRC metastasis is a critical and unresolved factor determining the overall survival time of patients.

The progression of CRC metastases is a complex process involving multiple signaling pathways [5]. In CRC, epithelial to mesenchymal transition (EMT) is associated with an invasive or metastatic phenotype. WNT/β-catenin, TGF-β, and EMT transcription factors such as ZEB1, ZEB2, and SNAIL are canonical CRC EMT regulators [6]. microRNAs (miRNAs) are also shown to play a critical role in regulating CRC metastasis by influencing the expression of key factors during metastasis [7]. A recent study also showed that miRNA could interact with other non-coding RNAs (ncRNAs), particularly circular RNAs, to regulate CRC metastasis [8].

The significance of long non-coding RNA (lncRNA) regulation in various diseases, including cancer, has been underlined [9, 10]. LncRNAs can regulate gene expression at two levels: transcriptional controls in the nucleus and mRNA stability, translation, and post-translational modifications in cytoplasm [11]. The fate and function of lncRNAs are determined by their direct interactions with RNA binding proteins and their indirect interactions with transcription factors and other proteins [12]. Alternatively, lncRNAs can operate as decoys for miRNAs, thus indirectly regulating mRNA expression [13].

Colorectal liver metastases (CRLM) are the most common type of CRC metastases [14]. Several lncRNAs have been shown to function in CRLM [15, 16]. However, none of these lncRNAs was CRLM-specific. The mechanisms of lncRNA regulation that have been established are more biased towards a specific gene, which may over estimate the impact of a single target gene.

To identify CRLM-related lncRNAs, a systematic analysis of lncRNAs differentially expressed between metastatic and primary CRC tissues was performed in this work. The function of CRLM1, which had not been described previously, was investigated. It was discovered that this lncRNA suppressed apoptosis and promoted the migration and metastasis of CRC cells, as well as liver metastases in Balb/C nude mice. ChIRP-seq and transcriptome analyses demonstrated that CRLM1 is associated with chromatin and acted as a powerful transcriptional regulator capable of altering the transcriptome toward metastasis. Additionally, ChIRP-MS research demonstrated an interaction between CRLM1 and hnRNPK protein, boosting nuclear localization and increasing promoter occupancy for transcriptional regulation linked with CRC metastasis. As a result, our study elucidates the potential role of CRLM1 in CRC metastasis and elucidates the molecular process by which tumor growth occurs. Our findings suggest that CRLM1 may be a prognostic factor for CRLM and a potential therapeutic target.

## Materials and methods

### Cell lines and cell culture

Procell (Wuhan, Hubei, China) provided the human CRC cell lines HCT116 and SW620. The cell line was authenticated using short tandem repeat (STR) analysis and checked for mycoplasma contamination by the Cell Bank, Type Culture Collection, and Chinese Academy of Sciences (CBTCCCAS). The STR analysis was carried out, as previously described [17].

HCT116 and SW620 cell lines were cultured in McCoy's 5A medium (Procell, China) containing 10% fetal bovine serum (BI) and 1% penicillin–streptomycin (Hyclone) at 37 °C in a humidified environment containing 5% CO_2_.

### Plasmids construction, short interference RNAs (siRNAs), and transfection

CRLM1 overexpression vector was designed, and human CRLM1 gene was cloned using reverse transcription and PCR amplification from total RNA taken from different cancer and non-cancer cell lines. DNA fragment matching the entire length of cDNA was purified from a gel (MinElute PCR Purification Kit, Qiagen, 28004) and then cloned into pTSB vector (TranSheepBio, China) using the hot fusion method (Vazyme Biotech Co., Ltd). Each primer comprises a fragment of gene-specific sequence and a 17–30 bp region from pTSB vector. The primers used for cDNA cloning were: CRLM1 F-Primer: ACCAGGTACAAAATCCCCTGG, R-Primer: TGGCTTGAACAATAAAAAGAGTTTATT. The pTSB vector was digested for 2 ~ 3 h at 37 °C by EcoRI (NEB, 3101S) and XhoI (NEBR0146V). The enzyme-digested vector was then put over a 1.0% agarose gel and purified using a Qiagen column kit (MinElute PCR Purification Kit, Qiagen, 28004). The insert fragment was then synthesized using PCR amplification. PCR insert was transferred to a PCR microtube for ligation (T4 DNA ligase, NEB, M0202V) with the ClonExpress® II One Step Cloning Kit (Vazyme, C112). The chemical transformation was used to introduce plasmids into an *Escherichia coli* strain. Cells were plated onto LB agar plates containing 1µL/mL ampicillin (sigma, 7177-48-2) and incubated at 37 °C overnight. Colonies were screened using colony PCR (28 cycles) and universal primers (located on the backbone vector). A DNA fragment encoding hnRNPK was obtained by PCR from HCT116 cell genomic DNA and subcloned into the pCDNA3.0 plasmid. Sanger sequencing was used to confirm the insert sequence. The siRNA for CRLM1#1 was 5′-GTGAAGAGAAGCTAAGGGATT-3′ while CRLM1#2 was 5′-GTGCTAACCTACAGAAGAATT-3′. The siRNA sequence for hnRNPK was 5′-CCCTAACACTGAAACCAATTT-3′. Lipofectamine 2000 Transfection Reagent (Invitrogen, Carlsbad, CA, USA, 11,668,019) was used to transfect HCT116 and SW620 cell lines with the siRNAs and the expression vector of hnRNPK according to the manufacturer’s protocols.

### Lentiviral production and stable transfection

The full-length RNAi sequences and antisense oligonucleotides were amplified by PCR and cloned into puromycin-selected lentiviral particles, which were constructed and packaged by Guangzhou RiboBio, Co., Ltd. The sequence of lentivirus as follows: sh-CRLM1: GTGCTAACCTACAGAAGAATT; sh-hnRNPK: TGCCAGTGTTTCAGTCCCAGA. To express CRLM1, a lentiviral pTSB-GFP-puro vector was employed. The lentiviral plasmids, and the packing plasmids pMD2.G and psPAX2 (TranSheepBio, China), were co-transfected into HEK293T cells using Lipofectamine 2000 according to the manufacturer’s protocol (Procell, China). At 72 h after transfection, lentiviruses were collected and utilized to transduce HCT116 and SW620 cells in a 24-well plate. Puromycin (2 µg/mL, Ameresco) was added 72 h after transduction to select drug-resistant cell types.

### Cell apoptosis, wound-healing assay, and transwell

Cell apoptosis was assessed using the BD Annexin V: FITC Apoptosis Detection Kit (BB-4101, BestBio, China). According to manufacturer's protocol, the cells were stained with Annexin V-FITC and propidium iodide (PI) to determine cell apoptosis rate using ACEA NovoCyteTM (ACEA Biosciences Inc, USA). CRC cells were seeded onto six-well plates and cultured to 90% confluence for the wound-healing assay. A sterile 200 μL pipette tip was used to scratch straight lines in the cell layer to produce wounds. The cells were washed twice with PBS and cultured in McCoy's 5A medium (Procell, China) supplemented with 1% serum media for 0 and 24 h at 37 °C. Wounds were seen at various time points within the scrape lines, and representative spots were marked and photographed in three separate fields. The wound closure was then seen using an inverted microscope (Nikon, Japan) at a magnification of 200×. The ImageJ software was used to determine the widths of wound gaps. Transwell chambers were used to conduct in vitro invasion experiments (3422, Corning). In the lower chambers, McCoy's 5A (Procell, China) containing 20% FBS (0.6 mL) served as a chemo-attractant, while the upper chambers were pre-coated with a thin layer of Matrigel (BD Biosciences, USA) diluted 1:3 with serum-free DMEM (Procell, China). 5 × 10^5^ cells in 0.2 mL of media with 1% serum medium were introduced to the inserts and incubated for 48 h at 37 °C and 5% CO_2_. Cells that remained on the upper membrane surface of the inserts were cleaned with a cotton swab. The total number of cells that migrated into the lower chamber was fixed in 4% paraformaldehyde for 20 min before staining with 0.1% crystal violet (Beyotime, China). The invasion cells were examined using an inverted microscope (Nikon, Japan) at a magnification of × 200.

### Animal experiments

SW620 cells were transduced with sh-CRLM1 alone, or CRLM1-OE alone or in combination with sh-hnRNPK, or corresponding negative controls (NCs), labeled as mock, CRLM1, sh-NC, sh-CRLM1, mock + sh-NC, CRLM1 + sh-NC, and CRLM1 + sh-hnRNPK groups. The cells were removed from the culture vessel and transferred to PBS for the in vivo liver metastasis experiment. 1 × 10^6^ SW620 cells were injected into the distal tip of the spleens of female Balb/c nude mice (4 weeks old) purchased from CAVENS (CAVENS Laboratory Lab, Changzhou, China). After 4 weeks, the animals were euthanized, and the livers were removed for study.

### Fluorescence in situ hybridization (FISH) and immunofluorescence (IF)

Analyses of fluorescence in situ hybridization Cy3 labeled FISH probes were designed and synthesized by Genepharma (Suzhou, China). Cells were cultured on glass coverslips for 20 min before being fixed with 4% paraformaldehyde and blocked for 30 min using Pre-hybridization Buffer. Cells were incubated with 2 μM CRLM1 FISH probe in Hybridization Buffer in the dark at 37 °C overnight, washed three times, blocked with 5% BSA, then incubated with anti-hnRNPK (11,426–1-AP, Proteintech), and Aluor-647 labeled the second antibody, finally incubated with DAPI (Genepharma, Suzhou, China) for detection for 15 min, and finally analyzed using a confocal (PerkinElmer, Olympus).

### RNA-seq

TRIZOL (Invitrogen) was used to extract total RNA from CRC cell lines. The sequencing library of each RNA sample was prepared using the PAKA RNA HyperPrep Kit according to the manufacturer’s protocol (Roche). The libraries were prepared according to the manufacturer's instructions for high-throughput sequencing using ABLife's IlluminaNext-Seq 500 platform for 151 nt pair-end sequencings (Wuhan, China).

### Chromatin immunoprecipitation (ChIP) assay

Approximately 1 × 10^7^ cells were cross-linked in 1% formaldehyde for 10 min before stopping with 0.125 M glycine for 5 min. The cross-linked cells were lysed in lysis buffer (1 × PBS, 0.1% SDS, 0.5% NP-40, and 0.5% sodium deoxycholate) and sonicated to generate DNA fragments ranging from 200 to 1000 bp. Immunoprecipitation of the protein-DNA complex was performed by incubating at 4 °C for 2 h with ChIP-grade Protein A/G Magnetic Beads conjugated with anti-hnRNPK antibody or IgG. The beads were washed five times with LiCl immune complex wash buffer (0.5 M LiCl), and once with TE buffer, 10 mM Tris (pH 8.0), and 1 mM EDTA (pH 8.0). After resuspending the beads in 100 µL elution buffer (100 mM NaHCO_3_, 1% SDS), they were reverse cross-linked overnight at 65 °C. DNA fragments were purified using phenol extraction and ethanol precipitation following sequential RNase A and proteinase K treatment. According to manufacturer's instructions, the libraries were constructed using ThruPLEX® DNA-seq kit (Rubicon Genomics) and then applied to the Illumina Next-Seq 500 platform by ABlife, Inc. for 151 nt pair-ends sequencings (Wuhan, China).

### Chromatin isolation by RNA purification (ChIRP)-MS and ChIRP-seq

RAPOligoDesigner was used to design probes and gene synthesis with biotin-labeled DNA. The ChIRP-MS and ChIRP-seq assays were carried out exactly as described previously [18, 19]. Approximately 2 × 10^7^ cells were cross-linked in 1% formaldehyde for 10 min before stopping with 0.125 M glycine for 5 min. The cross-linked cells were lysed in lysis buffer (50 mM Tris–Cl pH 7.0, 10 mM EDTA, 1% SDS) and sonicated to generate 100–500 bp DNA fragments. Chromatin is diluted in a hybridization buffer two times its volume (750 mM NaCl, 1% SDS, 50 mM Tris7.0, 1 mM EDTA, 15% formamide). The DNA oligonucleotides were designed as probes to detect the lncRNA-CRLM1. As a negative control, LacZ from *E. coli* was used. Additional file 7: Table S1 displays the probe sequences. In HCT116 cells, probe sequences were used to capture lncRNA-CRLM1 and LacZ. To 3 mL of diluted chromatin, 100 pmol of probes were added, then mixed by end-to-end rotation at 37 °C for 4 h. Streptavidin magnetic C1 beads were washed three times in nuclear lysis buffer before being transferred into the reaction with 1 mL of sample and mixed for another 1 h at 37 °C. Magnets (Invitrogen) were used to capture beads: biotin-probes: RNA: chromatin adducts, which were then washed five times with wash buffer (2 × SSC, 0.5% SDS, v/v to original bead volume). After the final wash, transfer half of the sample to a new tube for MS analysis. For DNA extraction and analysis, use a different substance. ChIRP-MS beads were resuspended in DNase buffer (100 mM NaCl and 0.1% NP-40), and protein was eluted at 37 °C for 30 min using a cocktail of RNase A, RNase H, and DNase I. After boiling for 10 min, the protein eluent was added with 5 × laemmli sample buffer. For western blots or MS, final protein samples were size-separated in bis–tris SDS-PAGE gels. Resuspend ChIRP-DNA beads in 150 µL DNA elution buffer (50 mM NaHCO_3_, 1% SDS), add RNase A and RNase H, and incubate at 4 °C for 30 min with shaking. Eluted chromatin was treated at 50 °C for 45 min with mixing. The DNA was then extracted with an equal volume of phenol/chloroform/isoamyl alcohol and precipitated with ethanol overnight at − 80 °C. According to manufacturer's instructions, high-throughput sequencing libraries were created from ChIRPed DNA using ThruPLEX® DNA-seq kit (Rubicon Genomics) and then applied to the Illumina Next-Seq 500 platform by ABLife, Inc. for 151 nt pair-end sequencings (Wuhan, China).

### quantitative real-time PCR (qRT-PCR) validation of differentially expressed genes (DEGs)

To validate RNA-seq findings, qRT-PCR was performed on a subset of DEGs and normalized to the reference gene GAPDH. Additional file 7: Table S2 contains information on the primer sequences. The primer table was used in the qRT-PCR experiment. For qRT-PCR, the same RNA samples used for RNA-seq were used. Denaturation at 95 ℃ for 10 min was followed by 40 cycles of denaturing at 95 ℃ for 15 s, annealing and extension at 60 ℃ for 1 min. For each sample, PCR amplifications were carried out in triplicate.

### RNA immunoprecipitation (RIP) assay

Cells were cross-linked with UV irradiation (254 nm) on the ice at 400 mJ/cm^2^. Place cross-linked cells in ice-cold wash buffer (1 × PBS, 0.1% SDS, 0.5% NP-40, and 0.5% sodium deoxycholate), then incubate with RQ I DNase for 3 min at 37 °C. For immunoprecipitation, the supernatant was treated for 2 h at 4 °C with Pierce™ Protein A/G Magnetic Beads coupled with anti-hnRNPK antibody or IgG. The beads were washed with wash buffer, high-salt wash buffer (250 mM Tris 7.4, 750 mM NaCl, 10 mM EDTA, 0.1% SDS, 0.5% NP-40, and 0.5% sodium deoxycholate) and 1 × PNK buffer (50 mM Tris, 20 mM EDTA, and 0.5% NP-40) twice, respectively. Proteinase K was used to digest the protein, and Trizol isolated the RNA. For RIP-qRT-PCR, a QuantStudio 6 Flex System with real-time detection was used (ABI). For qRT-PCR experiment, a primer table was employed. Additional file 7: Table S3 shows gene-specific PCR primer pairs.

### Western blot

The nuclear and cytoplasmic fractions of HCT116 and SW620 cells transfected with CRLM1-OE were prepared using the nuclear and cytoplasmic extraction kit (Thermo, Waltham, MA) according to manufacture's instructions. HCT116 and SW620 cells transfected with or without CRLM1-OE and/or si-hnRNPK were lysed with RIPA lysis buffer (R0010, Solarbio, China) supplemented with protease inhibitors (Roche). Protein concentrations were measured using a BCA kit (Pierce, Rockford, IL). Then, the total protein was divided using 10% sodium dodecyl sulfate polyacrylamide gel electrophoresis (SDS-PAGE), followed by transfer to polyvinylidene fluoride (PVDF) membrane (Merck Millipore, Billerica, MA). For blocking the membrane, 5% skimmed milk was applied for 1 h, followed by primary antibodies against hnRNPK (Proteintech), BBC3 (Sigma-Aldrich), SMAD6 (ABclonal), BMP7 (Proteintech) and SMURF2 (ABclonal) overnight at 4 °C. Following the PVDF membrane's washing with TBST, it was incubated for 2 h with the corresponding secondary antibody. The proteins were identified using Pierce SuperSignal West Pico Chemiluminescent Substrate (Termo Fisher, Waltham, MA), following the manufacturer's instructions. Internal references were HistoneH3 and GAPDH antibody.

### Statistical analysis

The R package factoextra (https://cloud.r-project.org/package=factoextra) was used to perform principal component analysis (PCA) to illustrate the clustering of samples with the first two components. After normalizing the reads in samples by TPM (Tags per Million), in-house-script (sogen) was used to visualize next-generation sequence data and genomic annotations. To do the clustering based on Euclidean distance, the heatmap package (https://cran.r-project.org/web/packages/pheatmap/index.html) in R was utilized. Gene set enrichment analysis (GEPIA) was used to perform a survival analysis for CRLM1 in The Cancer Genome Atlas (TCGA) CRC data [20]. GraphPad Prism 9.0 was used for statistical analysis (GraphPad Software Inc., CA, USA). The mean and standard deviation express the data (Standard Deviation). The two-tailed Student's t-test, ANOVA, or χ^2^ test were used to compare two groups. *P* < 0.05 was regarded as statistically significant, *P < 0.05, **P < 0.01, ***P < 0.001.

All other materials and methods can be found in Additional file 1.

## Results

### CRLM1 is overexpressed in colorectal liver metastases and associated with poor survival

**Fig. 1:**
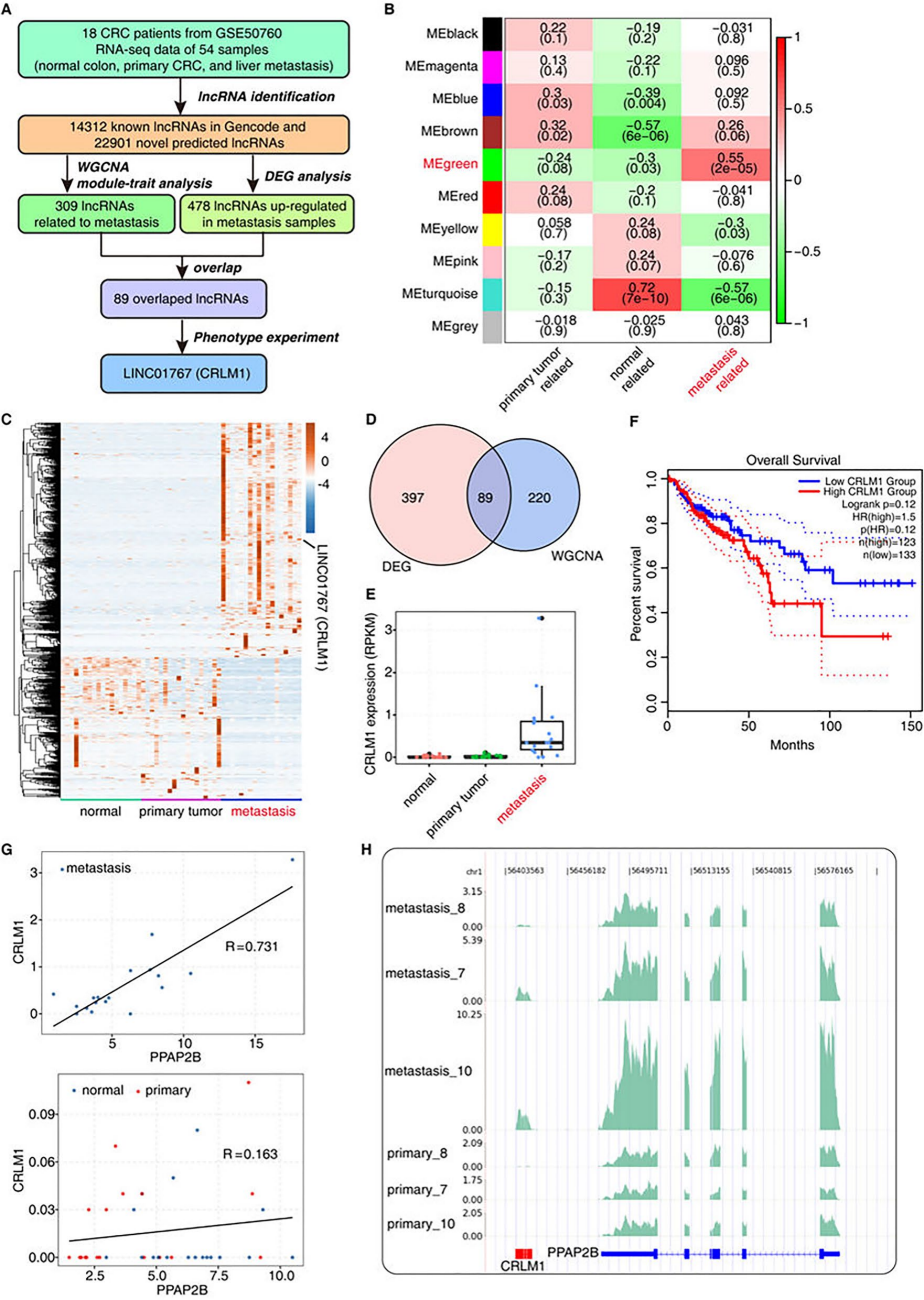
CRLM1 is overexpressed in colorectal liver metastases and associated with poor survival. **A** Strategy of lncRNA CRLM1 identification by WGCNA and DEG analysis. **B** Module-trait association. Each row corresponds to a module and each column corresponds to a specific trait: normal related, primary tumor related, and metastasis tumor related. The color of the cell indicates the correlation coefficient between the module and trait, dark red color indicates high degree of positive correlation, and dark green indicates high degree of negative correlation between each module and trait. The numbers in the cell indicate the module-trait correlation value (top) and *p* value (below). **C** The unsupervised hierarchical clustering heatmap of 54 samples based on DElncRNAs between metastasis group and primary group. **D** Venn diagram of up-regulated lncRNAs in metastasis (vers. Primary) and the most metastasis-related module (MEgreen) detected by WGCNA. **E** Boxplot of expression level of CRLM1 in normal samples, primary and metastasis tumor samples. **F** Kaplan–Meier analysis for overall survival of patient with high or low CRLM1 in the colorectal cancer from TCGA dataset. **G** Expression of CRLM1 and the adjacent gene PPAP2B is positive correlated in metastasis. **H** Visualization of CRLM1 and the adjacent gene PPAP2B

To conduct a thorough examination of the lncRNA expression landscape in CRLM tissues, 54 whole transcriptome sequencing data (RNA-seq) from 18 CRC samples, comprising normal colon, primary CRC, and liver metastasis tissues, were downloaded [21]. To discover lncRNAs linked with CRC metastasis, an analysis pipeline integrating different screening methodologies was used (Fig. 1A). The RNA-seq data was used to predict novel lncRNAs and lncRNA functions (Additional file 2: Fig. S1A). By overlapping the expressed lncRNAs in these three groups, it was discovered that there was a high overlap for both known and novel lncRNAs (Additional file 2: Fig. S1B). The first component of the principal component analysis (PCA) of the expressed lncRNAs and mRNAs revealed a significant difference between normal and tumor tissues. Still, the second component revealed a clear but not very different split between primary and metastatic tissues (Additional file 2: Fig. S1C). Weighted gene co-expression network analysis (WGCNA) was used to investigate metastasis-associated lncRNAs, and it discovered a co-expression module (MEgreen, including 309 lncRNAs) that was strongly related to metastatic tissues in a positive manner but not with normal/primary tissues (Fig. 1B, Additional file 2: Fig. S1D, E, and Additional file 7: Table S4). Concurrently, DEG analysis was performed to identify lncRNAs that were abnormally up-regulated in metastatic tissues (Fig. 1C, Additional file 2: Fig. S1F, and Additional file 7: Table S5), yielding 486 lncRNAs. The dysregulated lncRNAs identified by WGCNA and DEG methods were overlapped to restrict the search targets, yielding 89 credible candidates (Fig. 1D).

Among these candidates, lncRNA-linc01767, later called colorectal liver metastasis one (CRLM1), was explored further. CRLM1 was expressed at much higher levels in metastatic tissues than in normal and primary tumor tissues (Fig. 1E). By categorizing CRC patients based on CRLM1 expression levels (TCGA dataset), it was discovered that CRLM1 expression was negatively associated with patient survival time (Fig. 1F). It is well known that lncRNAs can control the expression of nearby genes in a cis-acting way. CRLM1 is found on chromosome 1 between the numbers 56,414,931 and 56,420,384. It has 4 exons that make up a 545-nt lncRNA and 3 short introns. The expression correlation between CRLM1 and its adjacent gene phospholipid phosphatase 3 (PPAP2B/PLPP3) was investigated, and it was discovered that these two neighboring genes were strongly correlated in metastasis tissues but not in normal or primary tissues (Fig. 1G, H), implying that CRLM1 may be an important regulator of PPAP2B expression. The relevance of phospholipid phosphatase in cancer metastasis is currently unknown.

### CRLM1 inhibits CRC cell apoptosis and promotes metastasis in vitro and in vivo

**Fig. 2:**
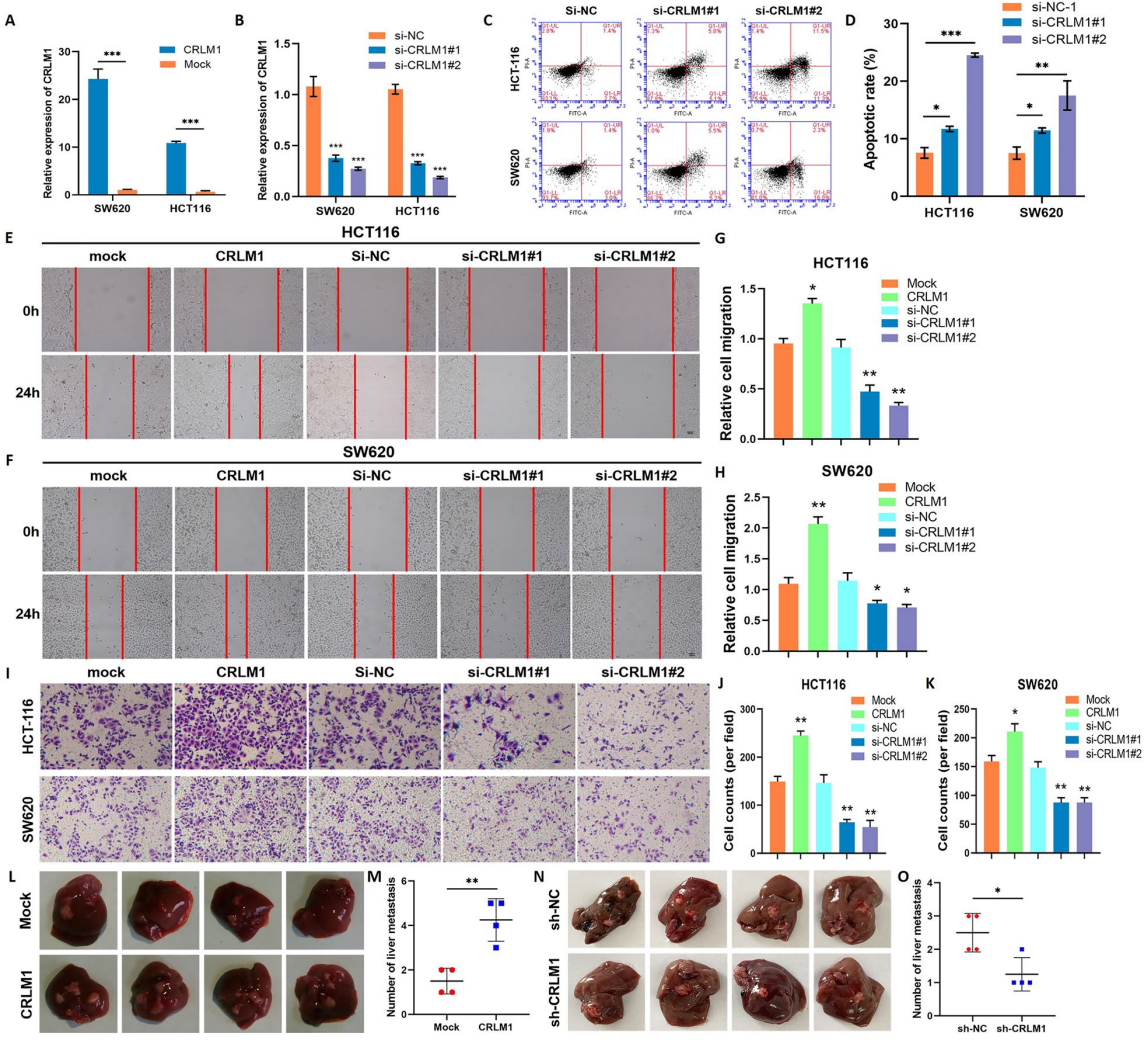
CRLM1 inhibits CRC cell apoptosis and promotes metastasis in vitro and in vivo. **A**, **B** The relative expression of CRLM1 was detected in HCT116 and SW620 cell lines after transfection with overexpressed CRLM1 plasmid (a) or si- CRLM1 (b) by qRT-PCR. **C**, **D** The apoptosis rate was analyzed by flow cytometry after downregulation of CRLM1 (**C**) and the quantification data (**D**). **E–H** The migratory ability of HCT116 (**E**) and SW620 (**F**) cell lines were detected by wound healing assays after overexpression or knockdown of CRLM1 (magnification, ×200, scale bar, 100 μm) and the quantification data (**G**, **H**). **I–K** Transwell assay was used to measure CRC cells invasion abilities after overexpression or knockdown of CRLM1 (magnification, ×200, scale bar, 100 μm) (**I**) and the quantification data (**J**, **K**). **L**–**O** Liver metastasis were taken from Balb/c nude mice injected with CRLM1-OE or sh-CRLM1 SW620 cells into the distal tip of the spleens for 4 weeks (n = 4 for each group). The images of liver metastasis (**L**, **N**) and the quantification data (**M**, **O**). Data are shown as mean ± SD, *P < 0.05, **P < 0.01, ***P < 0.001

CRLM1 overexpression (CRLM1-OE) plasmids and siRNAs targeting CRLM1 were transfected into HCT116 and SW620 cell lines to further investigate the biological activities of CRLM1 in CRC cells. Using qRT-PCR, the researchers discovered that the expression of CRLM1 was significantly up-regulated or down-regulated in CRC cells transfected with the corresponding vectors or siRNAs (Fig. 2A, B). Apoptosis was shown to be considerably enhanced in CRLM1 knockdown cells (Fig. 2C, D). To examine the migratory and invasive characteristics of CRLM1 overexpression or knockdown CRC cells, wound-healing and transwell assays were performed. The results showed that up-regulation of CRLM1 considerably boosted the migratory and invasive abilities of CRC cells, whereas down-regulation of CRLM1 significantly reduced them (Fig. 2E–K). To investigate the role of CRLM1 in CRC metastasis in vivo, we implanted stable CRLM1-OE or sh-CRLM1 SW620 cells into the distal tip of the spleens of Balb/c nude mice. Compared to the control group, the mice in the CRLM1-OE group developed more widespread liver metastasis (Fig. 2L, M), while the sh-CRLM1 group showed reduced liver metastasis (Fig. 2N, O). These findings added to evidence that CRLM1 inhibits CRC cell apoptosis and increases metastasis in vitro and in vivo.

### CRLM1 regulates the expression of CRC metastasis-related genes

**Fig. 3:**
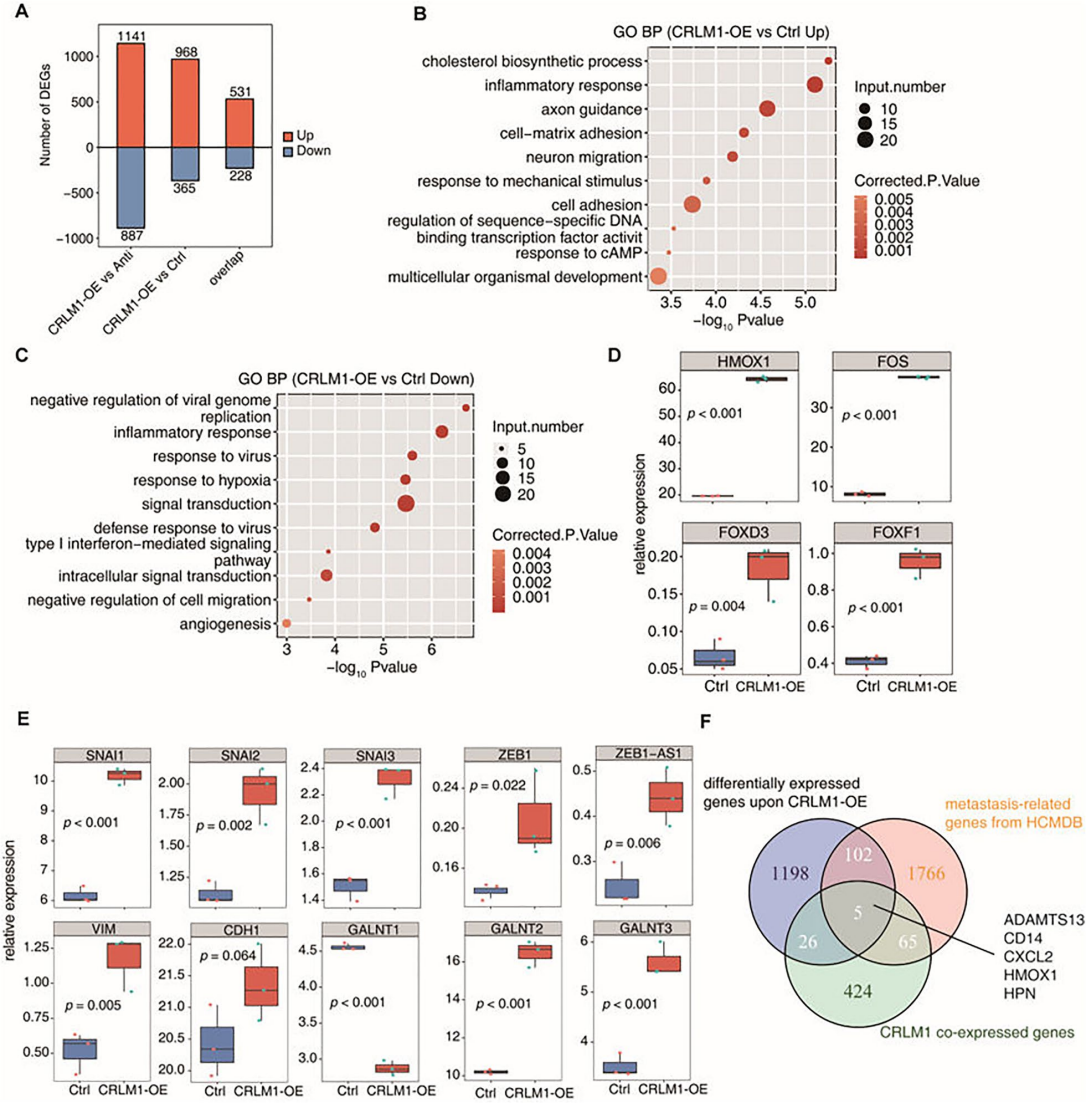
CRLM1-OE converts the CRC cell transcriptome to the metastasis state. **A** The number of DEGs among different groups. The number of up-regulated and down-regulated DE lncRNAs was showed in bar plot. “overlap” indicates the co-regulated genes between CRLM1 vs Anti and CRLM1 vs Ctrl. **B**, **C** The top 10 representative GO Biological Process terms of up- (**B**) and down-regulated genes (**C**) between CRLM1-OE and Ctrl. **D** Boxplots showing expression status of 4 differentially expressed TFs comparing CRLM1-OE with Ctrl samples. **E** Boxplots showing expression status of 10 EMT-related genes comparing CRLM1-OE with Ctrl samples. **F** Venn diagram of differentially expressed genes upon CRLM1-OE, metastasis-related genes from HCMDB database and CRLM1 co-expressed genes

A whole transcriptome sequencing experiment (RNA-seq) was done on three biological replicates of HCT116 cells transfected with CRLM1-OE, antisense-OE, and free plasmid control to reveal the underlying molecular mechanism for CRLM1 enhanced metastasis of CRC to the liver. CRLM1-regulated genes were identified utilizing DEG analysis with antisense-OE control and free plasmid control. The comparison revealed 1141 and 968 up-regulated genes for the antisense and free plasmid controls, respectively, and 887 and 365 down-regulated genes, with the bulk of these DEGs shared (Fig. 3A and Additional file 7: Table S6). The DEGs that emerged from the comparison with the free plasmid control were studied further.

Several cancer metastasis-related gene ontology (GO) biological process (BP) terms, including inflammatory response, cell–matrix adhesion, neuron migration, and cell adhesion, were discovered by functional analysis of CRLM1 up-regulated genes (Fig. 3B and Additional file 3: Fig. S2A). CRLM1 down-regulated genes were also enriched in cancer-related BP terms such as inflammatory response, hypoxia response, negative regulation of cell migration, and angiogenesis (Fig. 3C and Additional file 3: Fig. S2B). Similar enriched GO BP terms were detected in up-regulated and down-regulated genes compared to CRLM1-anti samples (Additional file 3: Fig. S2C, D). These findings showed a link between CRLM1 and cancer metastasis.

DEGs induced by CRLM1 were also enriched in transcription factors. Transcriptional factors such as HMOX1, FOS, FOXD3, and FOXF1 that have been up-regulated may contribute to the metastatic transcriptome formed by CRLM1-OE (Fig. 3D). Additionally, CRLM1 boosted the expression of classic EMT transcription factors, including Snails, ZEB1 and ZEB1-AS, which positively regulates ZEB1 expression in CRC cells, which should be done contribute significantly to the metastatic transcriptome as well. The EMT marker vimentin expression was greatly elevated, whereas the epithelial marker CDH1 (E-cadherin) expression was not altered. Recently, it was shown that GALNT1 promotes metastasis. Its expression is regulated by the lncRNA SNHG7 [15]. However, it was revealed that CRLM1 drastically lowered GALNT1 expression while increasing GALNT2 and GALNT3 expression (Fig. 3E). To verify the results of RNA-seq, qRT-PCR was performed to detect the expression of above genes in HCT116 cells transfected with CRLM1-OE. The results showed that HMOX1, FOS, FOXD3, FOXF1,SNAI1, SNAI2, SNAI3, ZEB1, ZEB1-AS1, VIM, GALNT2 and GALNT3 mRNA level in CRLM1-OE cells was significantly higher than in control cells, whereas GALNT1 was significantly lower. And there was no significant difference in CDH1 expression between CRLM1-OE cells and control cells (Additional file 3: Fig. S2E, F). These findings were similar to the RNA-seq results.

A co-expression study of 54 RNA-seq datasets revealed that CRLM1 expression was linked with 520 genes, 31 of which overlapped with CRLM1-OE DEGs in CRC cells. The CRLM1-regulated and CRLM1-coexpressed genes were compared to metastasis-related genes (1938) in the human cancer metastasis database (HCMDB) [22], yielding 107 and 70 overlapped genes, respectively (*p* = 0.002 and 4.48123e-10, hypergeometric test, Fig. 3F). These three datasets shared only five genes, including ADAMTS13, CD14, CXCL2, HMOX1, and HPN (Fig. 3F). Heatmap analysis revealed that these five genes were overexpressed in metastatic samples (Additional file 3: Fig. S2G), while three (ADAMTS13, CD14 and HMOX1) were up-regulated and two (CXCL2, and HPN) were down-regulated in CRC cells by CRLM1-OE (Additional file 3: Fig. S2H). Consistent with the positive correlation between CRLM1 and its neighboring gene PPAP2B in patients, it was demonstrated that CRLM1 boosted PPAP2B expression in vitro (Additional file 3: Fig. S2I), indicating that CRLM1 has a regulatory effect on PPAP2B expression.

### CRLM1-chromatin binding is weakly associated with CRLM1-regulated gene expression

**Fig. 4:**
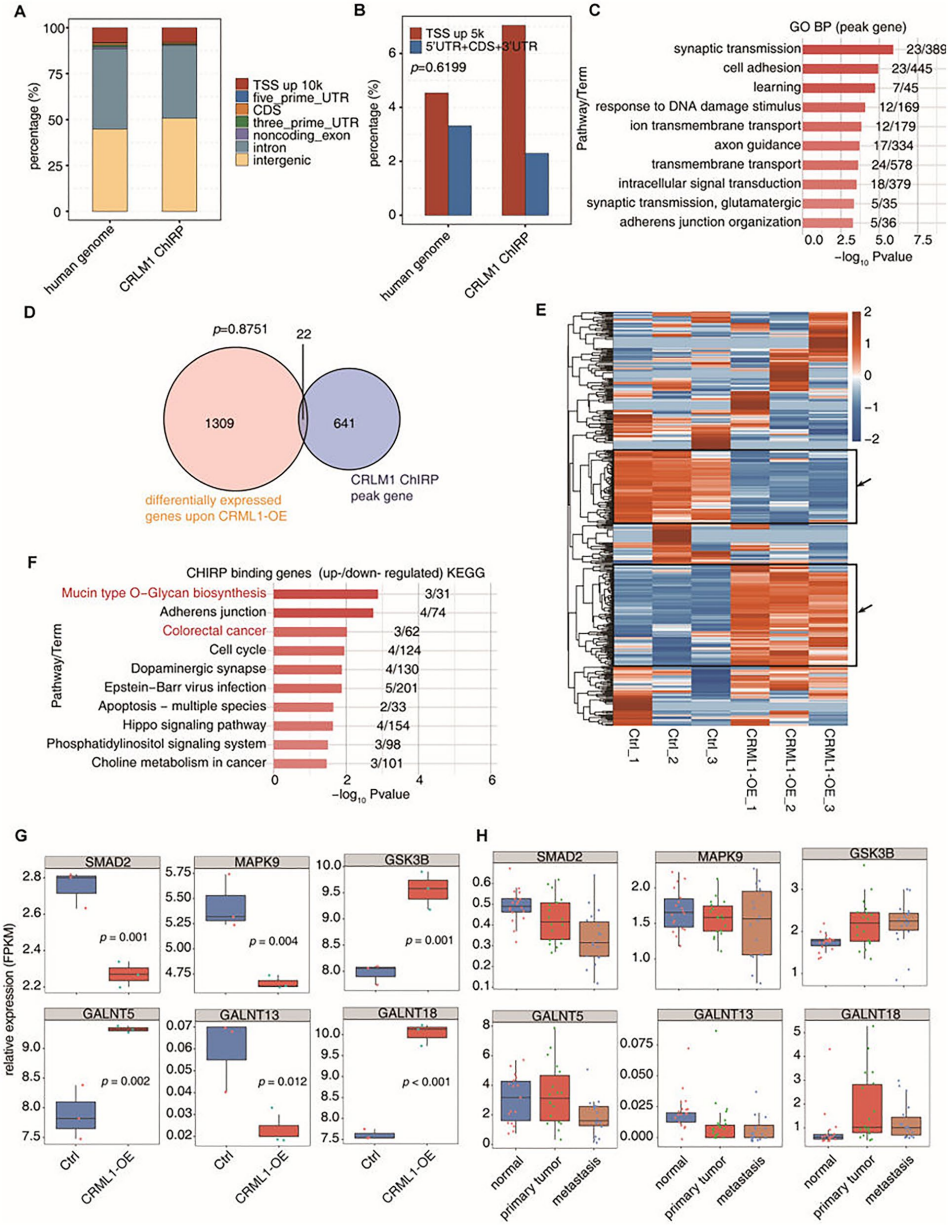
CRLM1–chromatin interaction and its effect on gene expression. **A** Percentage of CRLM1-bound peaks in different genomic regions. **B** Percentage of CRLM1-bound peaks in 5 kb of TSS upstream was compared to that in gene body exclude intron region. **C** The top 10 representative GO Biological Process terms of all CRLM1 binding genes. **D** Venn diagram of CRLM1 binding genes by ChIRP-DNA and differentially expressed genes upon CRLM1-OE. **E** The unsupervised hierarchical clustering heatmap showing the expression level of CRLM1-bound genes by ChIRP-DNA from CRLM1-OE and Ctrl samples. **F** The top 10 representative GO Biological Process terms of CRLM1-bound genes which were regulated by CRLM1-OE (genes in black box of **D**). **G** Boxplots showing expression status of six CRLM1-bound genes which were regulated by CRLM1-OE genes from CRLM1-OE and Ctrl samples. **H** Boxplots showing expression status of six CRLM1-bound genes which were regulated by CRLM1-OE genes from 54 GEO samples

Numerous lncRNAs regulate gene expression through their association with specific chromatin sites. To elucidate how CRLM1 regulates gene expression in HCT116 cells, the ChIRP approach was used to identify putative CRLM1–chromatin interaction sites by sequencing the chromatin DNA fragments associated with CRLM1 (ChIRP-seq) (Additional file 4: Fig. S3A). One thousand three hundred and one peaks were obtained after aligning the quality-filtered data to the human genome and calling Model-based Analysis of ChIP-Seq 2 (MACS2) peaks (Fig. 4A and Additional file 7: Table S7). When compared to the genomic length distribution, there were more CRLM1 binding sites (peaks) in the TSS upstream region than in the gene body (excluding the intron) (Fig. 4B). Six hundred sixty-three genes having CRLM1 peaks in the gene body region or the upstream 5 kb region were identified. Notably, CRLM1-associated genes were also considerably enriched in biological processes associated with metastasis, such as cell adhesion and response to DNA damage stimuli (Fig. 4C and Additional file 4: Fig. S3B).

However, only 15 and 7 of these genes overlapped with CRML1 up-and down-regulated genes (Fig. 4D). These findings imply that CRLM1 binding and CRLM1-regulated gene expression are not well correlated. The global expression pattern of genes containing one or more CRLM1-associated chromatin locations was studied using heatmap clustering. Around half of the CRLM1-associated genes exhibited distinct CRLM1-regulated gene expression patterns, with more up-regulated genes than down-regulated genes in CRLM1-OE samples (black frames, Fig. 4E). The activities of these CRLM1-associated genes were also investigated. They were found to be strongly correlated with cancer metastasis-related terms, including adhesion junction, cell cycle, apoptosis, and choline metabolism in cancer (Fig. 4F). Notably, these genes were significantly enriched in mucin-type O-glycan biosynthesis and CRC (Fig. 4F). Changes in O-glycan biosynthesis were previously observed in human CRC tissues [23]. The three CRC genes were SMAD2, MAPK9, and GSK3B. When the expression levels of these genes and three mucin-type O-glycan biosynthesis genes, GALNT5, GALNT13, and GALNT18, were plotted, we discovered that the expression change for all of these CRLM1-binding genes was low or negligible (Fig. 4G). qRT-PCR was performed to verify the mRNA expression of these genes of HCT116 cells transfected with CRLM1-OE. Similar results were obtained from qRT-PCR (Additional file 4: Fig. S3C). Interestingly, the expression patterns of these genes were largely similar in patient metastatic tissues (Fig. 4H) to those in CRLM1-OE CRC cells, implying the functional significance of the CLRM1 binding-associated gene expression changes, albeit minor. It was observed that the link between CRLM1 and chromatin was poor. Except for CRLM1 itself, most of the CRLM1 binding sites had poor ChIRP-seq signals (Additional file 4: Fig. S3D–F).

### CRLM1 physically interacts with hnRNPK and promotes its nuclear localization

**Fig. 5:**
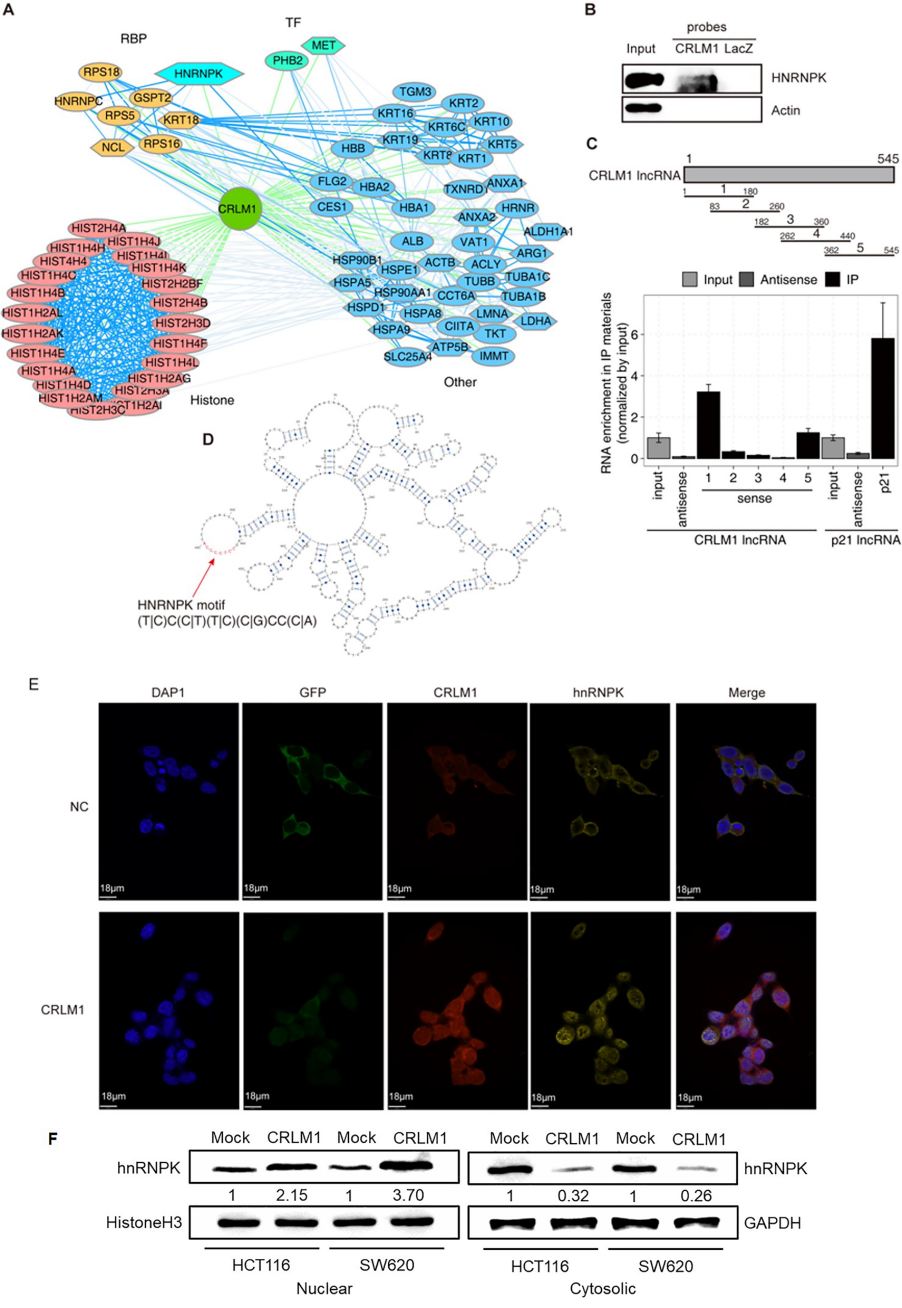
CRLM1 interacts with hnRNPK and promotes its nuclear localization. **A** CRLM1–protein interaction network. Histone, RNA binding proteins (RBP), transcriptional factors (TF) and other proteins are grouped separately. Hexagon indicates the metastasis-related genes from HCMDB database. The protein association networks were constructed using STRING. **B** Western validation of HNRNPK protein retrieved by CRLM1. **C** hnRNPK RIP-qPCR. **D** Predicted second structure of CRLM1 which contains hnRNPK binding motif at 403 to 410. **E** Localization of CRLM1 and hnRNPK in HCT116-NC and HCT116-CRLM1 cells by fluorescence in situ hybridization and immunofluorescence. Blue represents DAPI staining of the nucleus, green represents GFP staining of the cytoplasm, red represents Cy3-labeled probe against CRLM1 RNA and yellow represents Aluor 647 against hnRNPK-antibody. **F** The hnRNPK protein from purified nuclear fractions and cytosolic fraction in HCT116 and SW620 cells transfected with CRLM1-OE was examined using western blotting

LncRNAs regulate cells function by interacting with proteins. ChIRP and mass spectrometry (ChIRP-MS) were used to identify proteins that interact with CRLM1. There were 76 proteins discovered in total (Additional file 7: Table S8). By clustering CRLM1-interacted proteins using STRING, it was discovered that CRLM1 interacts preferentially with a large number of histone proteins and a few RNA binding proteins (RBPs), shedding light on the potential processes by which CRLM1 interacts with chromatin (Fig. 5A). The transcriptional regulatory role of RBPs was discovered using a large-scale genome-wide chromatin immunoprecipitation (ChIP) approach. Among the RBPs that interact with CRLM1, heterogeneous nuclear ribonucleoprotein K (hnRNPK) has been investigated for decades as a DNA-binding transactivator that recruits transcription factors that promote cancer development and metastasis. As a result, it was suggested that CRLM1 might interact with hnRNPK to perform its metastasis-promoting functions.

Western blotting was used to confirm the physical interaction between CRLM1 and hnRNPK using protein components recovered from the CRLM1 ChIRP experiment. hnRNPK was found in the CRLM1 ChIRP sample but not in the LacZ negative control sample, and both ChIRP samples were devoid of ACTIN (Fig. 5B). To identify the unique 483 fragment capable of engaging with hnRNPK, we used an enhanced RNA immunoprecipitation (RIP) assay employing antibodies against hnRNPK to capture its associated RNA fragments. PCR amplification of the related CRLM1 RNA resulted in five distinct truncation segments with approximately 80 ~ 100 nt overlap between neighboring fragments (Fig. 5C). As a positive control, a well-characterized lncRNA-P21 was chosen that interacts with hnRNPK [24]. As negative controls, antisense strands were amplified. The results of qRT-PCR indicated that the first fragment (position 1–180) had the strongest binding signal, but the second fragment (position 83–260) had a very weak signal, implying a significant binding site in an 82-nt CRLM1 fragment positioned at the 5′ end (Fig. 5C). Similarly, a study of the binding signal difference between the fifth (position 362–545) and fourth (position 262–440) CRLM1 fragments showed the presence of a second strong binding site for hnRNPK in a 105-nt CRLM1 fragment positioned at the 3′ end of CRLM1 (position 441–545). Previous research established that hnRNPK binds to C-rich motifs and that this binding is connected with lncRNA nuclear enrichment [25, 26]. The C-rich and Alu elements in CRLM1 sequences were subsequently examined, and a putative hnRNPK binding site was identified in a large loop located at the 3′ end (position 483 ~ 490, Fig. 5D), consistent with RIP-qPCR results.

To further investigate the physical interaction between CRLM1 and hnRNPK, fluorescence in situ hybridization (FISH) tests were performed to confirm their cellular location. Under negative control, CRLM1 was both nuclear and cytoplasmic, whereas hnRNPK was more cytoplasmic than the nucleus. However, it was discovered that CRLM1 and hnRNPK were mostly localized in the nucleus with some colocalization under the CRLM1-OE condition. Surprisingly, hnRNPK's cytoplasmic distribution was decreased (Fig. 5E). To verify the results of FISH experiment, western blotting was performed to analyse the nuclear/cytosol fractionation and quantification of CRLM1-dependent hnRNPK in HCT116 and SW620 cells transfected with CRLM1-OE. The results showed that CRLM1-OE cells exhibited significantly higher levels of nuclear hnRNPK protein, compared with the control cells, as well as cytosolic hnRNPK protein level was significantly lower than in control cells (Fig. 5F). This finding was consistent with the FISH results. Taken together, our results indicate that CRLM1 interacts with and supports the nuclear localization of hnRNPK.

### CRLM1 strongly promotes hnRNPK occupancy at promoter regions to regulate the expression of genes

**Fig. 6:**
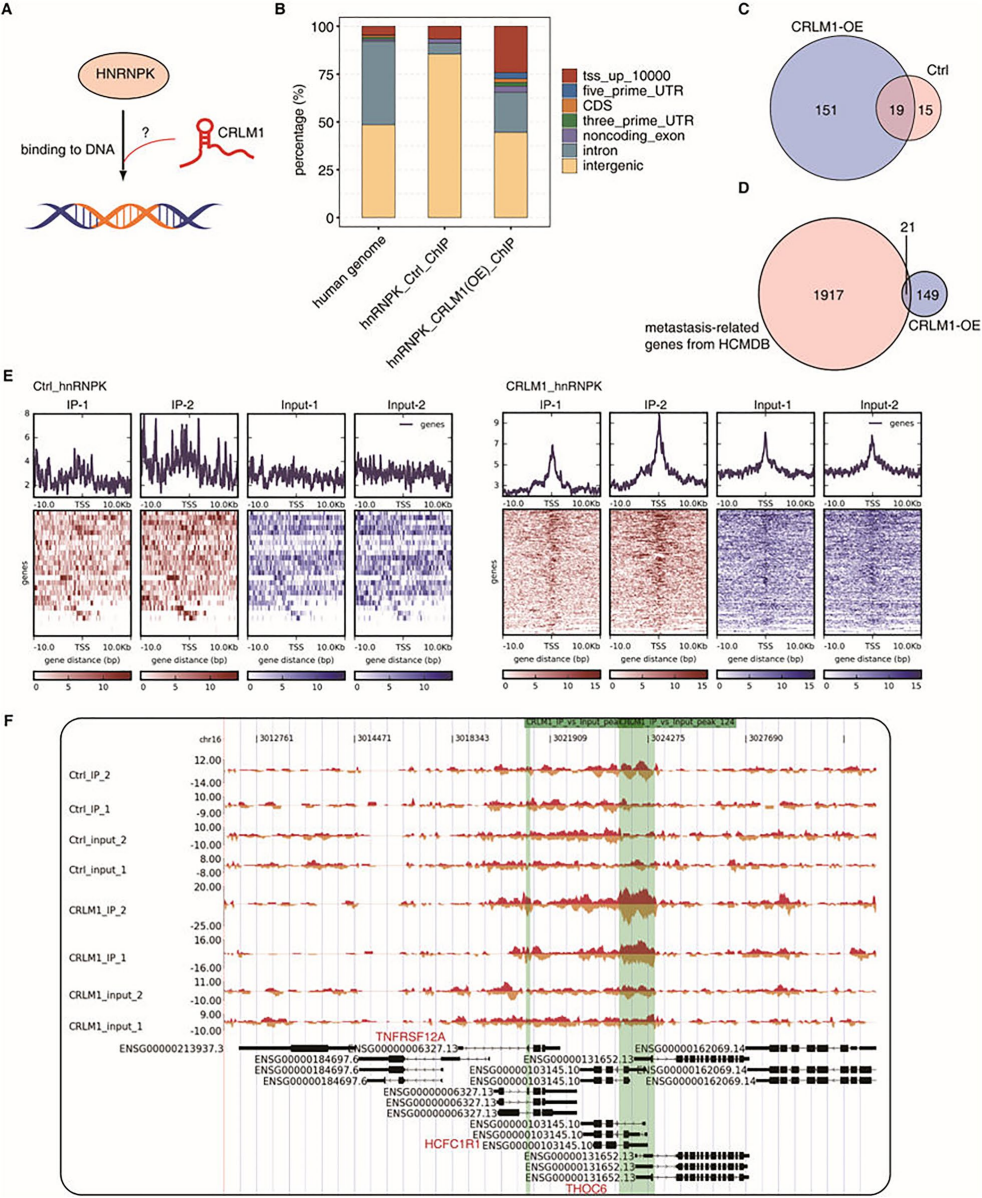
CRLM1 promotes hnRNPK promoter occupancy. **A** Illustration of the working model. **B** Genic regions of hnRNPK binding peaks without CRLM1 (Ctrl) and with CRLM1-OE. **C** Venn diagram of hnRNPK binding genes without CRLM1 and with CRLM1-OE. **D** Venn diagram of hnRNPK binding genes with CRLM1-OE and metastasis-related genes from HCMDB database. **E** Global visualization of hnRNPK signals in Ctrl (left) and CRLM1 (right) around gene TSS by ngs.plot. **F** Example for CRLM1-promoted hnRNPK binding profiles

Given that CRLM1 may interact with hnRNPK and increase its nuclear localization in trans, the question is how CRLM1 affects the hnRNPK-chromatin relationship (Fig. 6A). To test this idea, chromatin immunoprecipitation and sequencing (ChIP-seq) for hnRNPK was done in HCT116 cells overexpressing hnRNPK under CRLM1-OE or control conditions. The confident hnRNPK ChIP peaks were mostly situated in intergenic regions, significantly more than the genomic fraction of intergenic areas (Fig. 6B, Additional file 7: Tables S9 and S10). Only 34 genes had at least one confident hnRNPK peak within their promoter regions (Fig. 6C), consistent with the low hnRNPK concentration in the nucleus. The CRLM1-OE condition significantly enhanced the hnRNPK peaks within 10 kb upstream of the TSS and in the gene body regions, decreasing intergenic regions. There are now 170 genes that contain at least one confident hnRNPK peak (Fig. 6B, C). One hundred and fifty-one were unique to the CRLM1-OE condition, 25 were duplicated with previously published hnRNPK-bound genes (1177) [27] (Additional file 5: Fig. S4A), and 21 were overlapped with metastasis-related genes (Fig. 6d). Functional enrichment analysis revealed that hnRNPK-bound genes were strongly enriched in apoptotic pathways and processes, as well as gene expression regulation and RNA metabolic processes (Additional file 5: Fig. S4B).

By plotting the peak reads surrounding transcriptional start sites, the effect of CRLM1 on triggering hnRNPK redistribution at transcriptional start sites was further investigated. It is demonstrated that the association between hnRNPK and promoter was poor in the absence of CRLM1 but dramatically increased in CRLM1 overexpression (Fig. 6E). Figure 6f and Additional file 5: Fig. S4C, D illustrate examples of CRLM1-mediated hnRNPK promoter occupancy, consistent with CRLM1-mediated nuclear localization of hnRNPK.

**Fig. 7:**
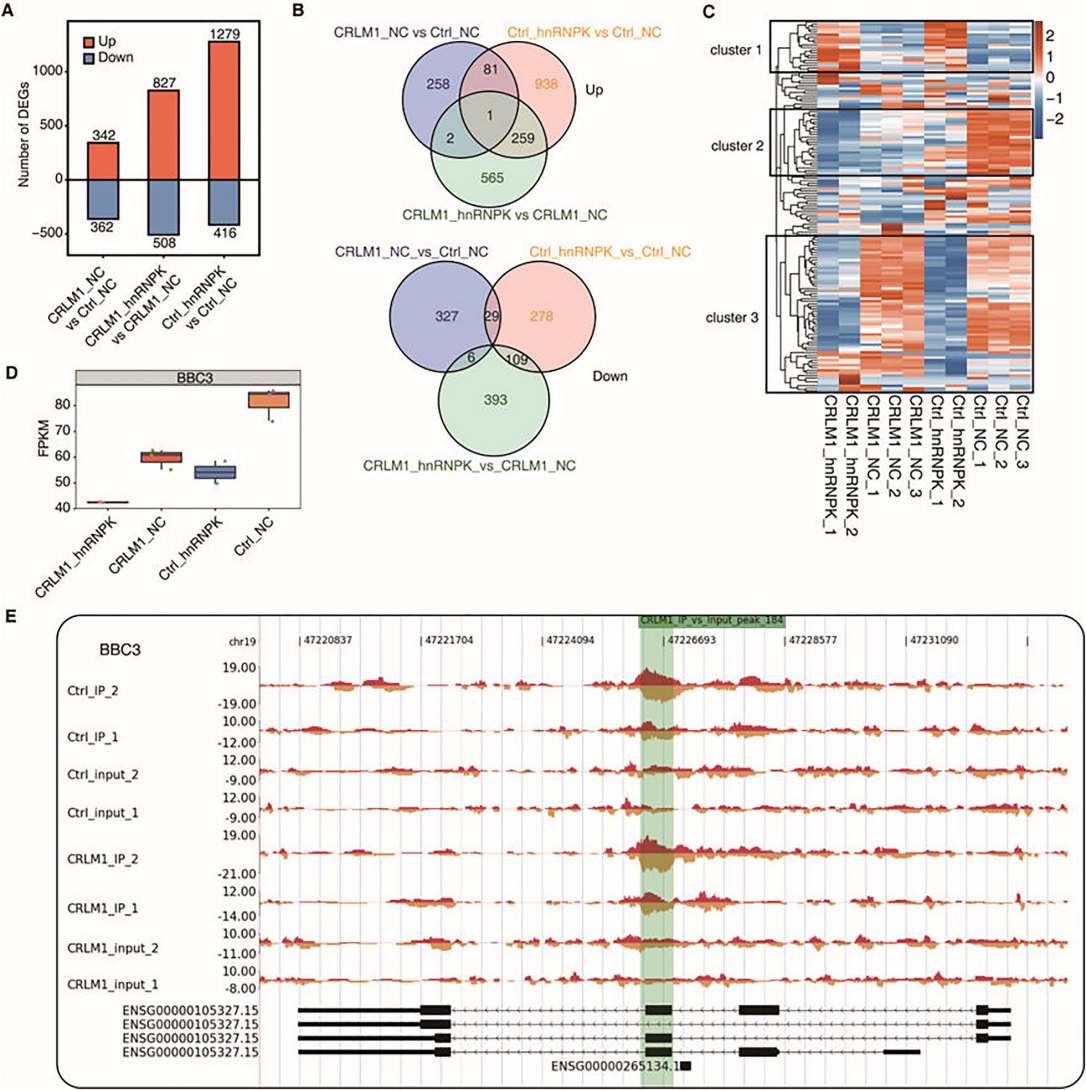
CRLM1 and hnRNPK co-regulates gene expression. **A** The number of DEGs among different groups. The number of up-regulated and down-regulated DE lncRNAs was showed in bar plot. **B** Venn diagram of co-up (up panel) and co-down (down panel) regulated genes among three groups in **A**. **C** The top 10 KEGG pathways of co-regulated DEGs between CRLM1 vs Ctrl and Ctrl_hnRNPK vs Ctrl. **D** The unsupervised hierarchical clustering heatmap of hnRNPK binding genes with CRLM1-OE. **E** Top 10 KEGG pathways of genes from cluster2 of **D**. **F** Box plot showing the expression profile of BBC3. **G** Visualization of hnRNPK binding profiles on BBC3

The nuclear localization and promoter binding of CRLM1-promoted hnRNPK are consistent with our hypothesis that CRLM1-hnRNPK regulates gene expression. To demonstrate this point, we overexpressed hnRNPK in HCT116 cells with and without stable CRLM1 overexpression (Additional file 6: Fig. S5A). Transcriptome sequencing (RNA-seq) was used to discover genes controlled by CRLM1 and hnRNPK. Pearson's correlation coefficients (PCCs) analysis revealed that samples containing the majority of hnRNPK-OE samples were well separated from other samples except for the third replicate, and the two outlier samples were excluded during the further study (Additional file 6: Fig. S5B). Functional enrichment analysis revealed that hnRNPK and CRLM1 co-regulated genes were strongly enriched in apoptotic processes, cell adhesion, as well as transcription regulation and metabolic processes (Additional file 6: Fig. S5C–F). Overexpression of either hnRNPK or CRLM1 resulted in significant alterations in gene expression, with more up-regulated genes than down-regulated genes (Fig. 7A, Additional file 7: Tables S11, S12, and S13). Surprisingly, many genes controlled by hnRNPK or CRLM1 overlapped (Fig. 7B), showing that they target the same collection of genes. When hnRNPK was overexpressed in cells expressing CRLM1, its ability to regulate gene expression was significantly reduced, particularly for up-regulated genes (Fig. 7A). This observation could be explained by the fact that genes regulated by hnRNPK are likewise regulated similarly by CRLM1. The hierarchical clustering heatmap revealed that hnRNPK overexpression altered the expression of the majority of hnRNPK-bound genes. Regardless of the degree of CRLM1 expression, hnRNPK overexpression promoted and suppressed the expression of genes in Cluster 1 and Cluster 3, respectively (Fig. 7C). In contrast, hnRNPK and CRLM1 inhibited the expression of genes in Cluster 2, and an additive inhibitory impact was observed for the majority of these genes (Fig. 7C). BBC3 was found to be a Cluster 2 gene (Fig. 7D, E). These findings demonstrate that CRLM1-mediated hnRNPK-chromatin association plays a significant role in gene expression regulation, with a subset of these genes exhibiting transcriptional suppression by both CRLM1 and hnRNPK.

### CRLM1-hnRNPK cooperatively regulates the expression of metastasis-related genes and promotes CRC cell metastasis

**Fig. 8:**
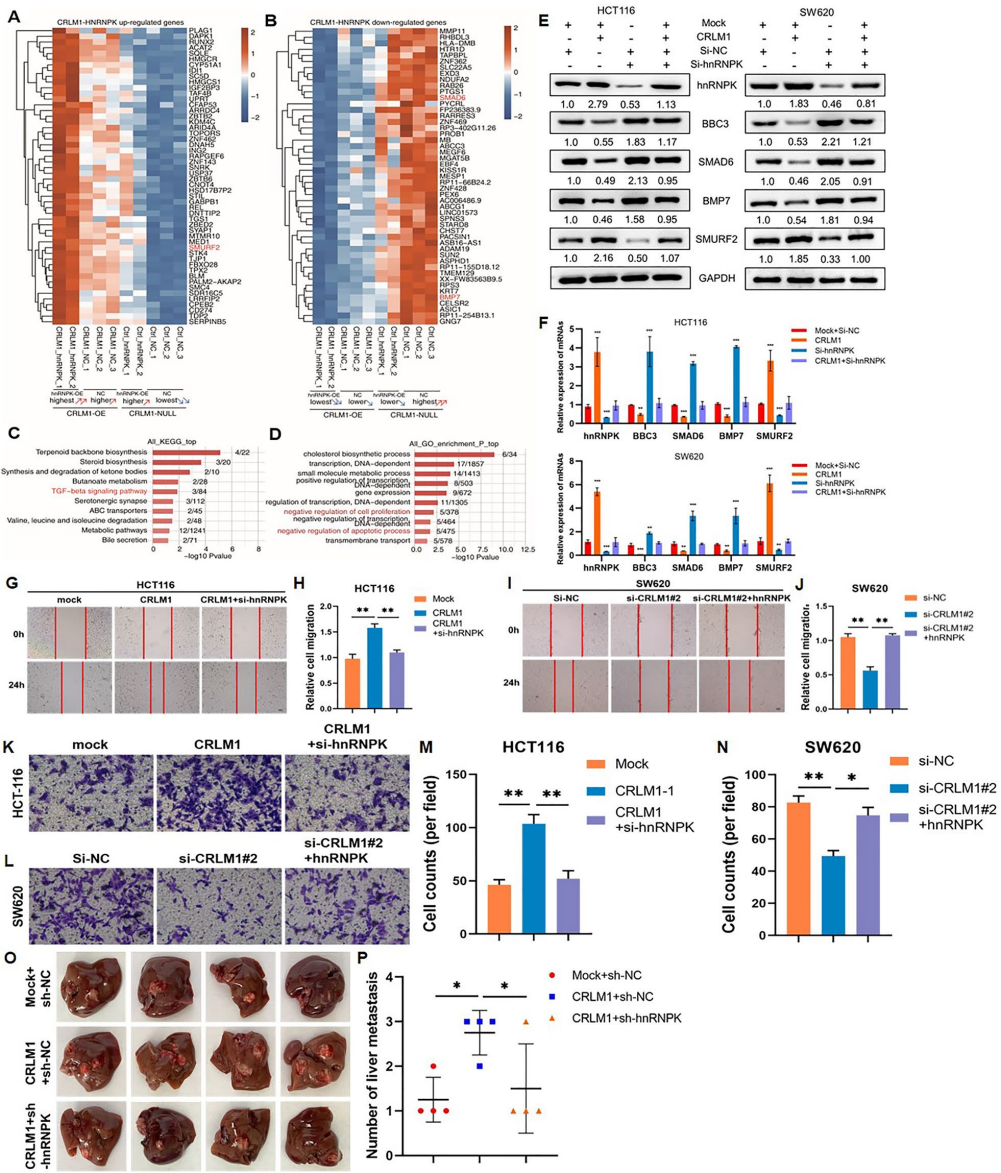
CRLM1-hnRNPK cooperatively regulates the expression of metastasis-related genes and promotes CRC cell metastasis. **A** CRLM1 and hnRNPK co-up-regulated genes. These genes were screened out through the criteria: fold change (FC) of CRLM1_hnRNPK vs CRLM1_NC ≥ 1.2, FDR ≤ 0.05; FC of CRLM1_NC vs Ctrl_NC ≥ 1.5, FDR ≤ 0.05; FC of Ctrl_hnRNPK vs Ctrl_NC ≥ 0.67 and ≤ 1.5. **B** CRLM1 and hnRNPK co-down-regulated genes. Criteria were same as (**A**) except the direction is opposite. **C**, **D** The top 10 KEGG pathways (**C**) and top 10 representative GO Biological Process terms (**D**) of all CRLM1 and hnRNPK co-regulated genes. **E** BBC3, SMAD6, BMP7 and SMURF2 protein expression in CRLM1-OE cells with hnRNPK knockdown by immunoblotting. **F** BBC3, SMAD6, BMP7 and SMURF2 mRNA expression in CRLM1-OE cells with hnRNPK knockdown by qRT-PCR. **G-J** The ability of CRC cell migration was detected by wound healing assay after transfection or co-transfection with corresponding vectors or siRNAs (magnification, ×200, scale bar, 100 m$\upmu$) (**G**, **I**) and the quantification data (**H**, **J**). **K–N** The invasion ability of CRC cells transfected or co-transfected with corresponding vectors or siRNAs was measured by transwell assay (magnification, ×200, scale bar, 100 μm) (**K**, **L**) and the quantification data (**M**, **N**). **O**, **P** hnRNPK knockdown inhibited liver metastasis of CRLM1-OE SW620 cells (n = 4 for each group). The images of liver metastasis (**O**) and the quantification data (**P**). Data are shown as mean ± SD, *P < 0.05, **P < 0.01, ***P < 0.001

The occupancy of the CRLM1-hnRNPK promoter by a collection of CRLM1-hnRNPK co-repressed genes represents the direct target of the CRLM1-hnRNPK-chromatin relationship. It was hypothesized that CRLM1-hnRNPK indirectly regulates other genes, which may play a role in metastasis and apoptosis regulation. It was discovered that CRLM1 and hnRNPK regulate the same genes cooperatively in the same direction. These genes were up-or down-regulated (52 and 49 genes, respectively) by both CRLM1 and hnRNPK. Double overexpression accentuated the up-or-down-regulation (Fig. 8A, B, Additional file 7: Tables S14 and S15). These CRLM1-hnRNPK co-regulated genes were found to enhance functional pathways such as the TGF- signaling pathway, a well-known mechanism directing EMT (Fig. 8C), and other cancer development activities such as negative regulation of cell proliferation and apoptosis (Fig. 8D). Three genes involved in TGF-β signaling pathways regulated by CRLM1-hnRNPK were down-regulated SMAD6 and BMP7, and up-regulated SMURF2. Additionally, when hnRNPK was knocked down, the stimulatory effect of CRLM1 on SMURF2 expression and the inhibitory effect on BBC3, SMAD6, and BMP7 expression was knocked down (Fig. 8E). The change in mRNA levels of these genes correlated with the change in protein levels (Fig. 8F). To determine if CRLM1 functions biologically via hnRNPK, rescue tests in CRC cells were done following co-transfection of si-hnRNPK or hnRNPK-OE with CRLM1-OE or si-CRLM1. The results indicated that knockdown hnRNPK could significantly reverse the pro-migratory and pro-invasion roles of CRLM1 up-regulation in CRC cells, whereas hnRNPK overexpression attenuated the inhibitory effects of CRLM1 down-regulation in CRC cells using wound healing and transwell tests (Fig. 8G–N). Additionally, in vivo rescue experiments were conducted to confirm that si-hnRNPK reversed the tumor metastasis promoter effect of CRLM1 in CRC cells. We found that knockdown of hnRNPK could effectively suppress liver metastasis of CRLM1-OE CRC cells in the liver metastasis model (Fig. 8O, P). These findings indicate that CRLM1 and hnRNPK work in concert to induce CRC cell metastasis.

## Discussion

Metastasis is a critical and unresolved factor affecting the overall survival time of patients with colorectal cancer. The liver is the most often encountered location of CRC metastasis [14, 28]. Patients with metastatic colorectal cancer (mCRC) continue to have a poor prognosis. Therapeutic target identification is critical to improving the curative condition of CRLM patients. Massive investigations of lncRNAs in recent years have identified several of their involvement in numerous diseases and their potential as diagnostic and prognostic biomarkers and druggable targets [9, 10]. The ability of a few lncRNAs to promote CRLM and act as poor prognosis markers has been reported in the last 2 years, even though none of these lncRNAs is CRLM-specific, and the mechanistic studies are skewed toward existing knowledge.

A systematic approach was used in our investigation to identify lncRNAs using previously published data comprising 54 transcriptomes from normal tissues, primary, and metastatic tumor tissues, resulting in the identification of a set of confident CRLM-related lncRNAs, including CRLM1. None of the recently described metastatic lncRNAs is included in this list. In the current genome version, CRLM1 is annotated as linc01767 (GRCH38). Unlike other recently discovered lncRNAs, the biological function of CRLM1 has not been established, most likely due to its unique expression pattern. A high CRLM1 expression level is closely associated with a low survival rate. It was proposed that these CRLM-related lncRNAs may constitute a novel class of prognostic and diagnostic markers for mCRC and a potential class of therapeutic targets. To assess the metastasis capability of CRLM1, it was demonstrated that up-regulation of CRLM1 inhibited apoptosis and increased metastasis in CRC cells in vitro and in vivo. Still, the downregulation of CRLM1 had the opposite effect. These findings demonstrate the metastatic capacity of CRLM1, whose expression is CRLM-related, and constitute the first biological studies on this lncRNA.

The mechanisms behind CRLM1-mediated apoptosis repression and metastasis induction were investigated using a genome-wide approach. RNA-seq investigation of the transcriptomes with CRLM1 overexpression demonstrates that CRLM1 has a significant effect on transcriptional regulation, as evidenced by over a thousand genes with a two-fold or more change in expression at a high level of statistical confidence. Among these, 107 have been identified as metastasis-related genes. The DEGs up-regulated by CRLM1 are highly enriched in cell–matrix adhesion, the master machinery of cell migration. Additionally, these genes are considerably enriched in the positive control of endothelial cell motility and cell-substrate adhesion. These unbiased transcriptome results significantly confirm the role of CRLM1 in enhancing CRC cell motility and invasion demonstrated in this investigation, as well as its suggested role in liver metastasis of CRC.

Furthermore, CRLM1 increased the expression of three snail transcription factors, SNAI1, SNAI2, SNAI3, ZEB1, and ZEB1-AS. The expression of EMT marker VIM was significantly increased. Because the change in expression of these important genes caused by CRLM1 overexpression was less than that of DEGs, they were not included in the functional clustering analysis. Combining CRLM1-regulated DEGs in CRC cells, CRLM1-coexpressed genes in CRC tissues and metastasis-related genes from the HCMDB database, five CRLM1-regulated metastasis genes of high clinical significance and confidence were identified: ADAMTS13, CD14, CXCL2, HMOX1, and HPN.

Numerous lncRNAs with nuclear localization are associated with chromatin. Typically, this association is thought to mediate chromatin remodeling in cis or trans by modulating the recruitment of chromatin modifiers [11]. Recent research indicates that this connection may recruit transcription factors to act as a mediator of transcriptional control. It was demonstrated that CRLM1 is mostly found in the nucleus. CRLM1 was found to be connected with chromatin in a ChIRP-seq assay. The 44 metastatic genes with CRLM1 peaks were enriched in cell adhesion function. Around half of the CRLM1 peak genes were related to either increased or decreased gene expression, including increased expression of colorectal cancer genes SMAD2, MAPK9, and GSK3β, as well as three members of GALNT family. SMAD2 is a well-studied metastasis gene, and MAPK9 and GSK3β also play a role in metastasis [29–31]. Recently, it was demonstrated that SNHG7 promotes CRC cell metastasis by up-regulating GALNT1 expression via miR-216b sponging [15]. By contrast, CRLM1 drastically decreased the expression of GALNT1 mRNA while considerably increasing the expression of GALNT2 and GALNT3, showing the presence of distinct mechanisms driving metastasis in CRC cells. However, except for the robust ChIRP-seq signals at CRLM1 gene body, the binding signals for most binding sites were extremely faint, and the extent of expression change for CRLM1 peak genes was generally minimal. As a result, it was proposed that CRLM1-chromatin link is weak, implying that its direct effect on gene expression is negligible. It is quite likely that CRLM1 collaborates with additional proteins to exert a strong regulatory effect on CRC cancer cell transcriptome.

Thus, ChIRP-MS investigations were used to detect CRLM1-interacting proteins in the nucleus and linked with chromatin, resulting in the discovery of hnRNPK and a huge number of additional proteins, including a large number of histone proteins. We noticed TUBB3 was one of the proteins related to CRLM1. TUBB3 promotes CRC cell migration and invasion, and its ubiquitination is required for metastasis via lncRNA RPPH1 [16]. The relationship of CRLM1 with TUBB3 warrants additional investigation to ascertain its contribution to the CRLM1 function in CRLM.

The hnRNPK contains three KH domains and binds both DNA and RNA [32]. The binding of HnRNPK to DNA can result in the transcriptional control of several cancer genes, including c-Myc, c-Src, and p53 target genes, by recruiting particular transcription factors, chromatin remodelers, and general transcription machinery components to promoters [33–35]. It was proven in this study that CRLM1 interacted with hnRNPK via its 5′ and 3′ terminal segments, with the 3′ fragment containing a characteristic C-rich pattern for hnRNPK binding. It demonstrated that a high amount of CRLM1 increases hnRNPK nuclear localization. Anti-hnRNPK ChIP-seq research demonstrated that CRLM1 significantly increased hnRNPK promoter binding. CRLM1 boosted hnRNPK-promoter association not only for genes co-regulated by CRLM1 and hnRNPK but also for genes regulated solely by hnRNPK, implying that some CRLM1 functions in promoting hnRNPK-promoter association are recessive in terms of gene expression regulation.

Additionally, it was found that CRLM1 and hnRNPK cooperated in regulating many genes, either directly or indirectly, including critical metastasis genes such as BMP7, SMAD6, and SMURF2 in the TGF-β signaling pathway [36–38]. These findings support the hypothesis that CRLM1 regulates metastasis by recruiting hnRNPK to the promoter regions of metastasis driver genes, where the secondary effect and further networking then amplify the signal. CRLM1-hnRNPK axis shares a major regulatory mechanism with the RBAT1-mediated transcription factor E2F3 [12].

## Conclusion

In summary, our findings indicate the presence of CRLM-related long noncoding RNAs and that one of these lncRNAs, CRLM1, not only predicts a poor prognosis but also inhibits apoptosis and promotes metastasis in vitro and in vivo. The mechanism by which CRLM1 regulates metastasis genes is attributed to its binding to hnRNPK, which promotes hnRNPK's nuclear localization and promoter occupancy, hence enabling CRLM1-hnRNPK co-regulation of metastatic genes. These findings contribute to our understanding of how lncRNA regulates transcription in trans and identify a prognostic marker and therapeutic target for CRLM.

## Supplementary Information

**Additional file 1.** Supplementary methods.

**Additional file 2: Fig. S1.** CRLM1 is overexpressed in colorectal liver metastases and associated with poor survival. **A** Illustration of bioinformatics analysis pipeline for the identification and functional annotation of lncRNA genes expressed in CRC samples. **B** Venn diagram of detected known lncRNA (left) and novel lncRNA (right) in normal, primary tumor and metastasis samples. At least two samples with RPKM ≥ 0.2 was considered to be detected in the group. **C** Principal component analysis (PCA) of samples based on all mRNAs (up) and lncRNAs (down). The samples were grouped by disease state and the ellipse for each group is the confidence ellipse. **D** A Heat map of module Eigengenes sorted by average linkage hierarchical clustering. **E** The unsupervised hierarchical clustering heatmap of 54 samples based on genes involved in MEgreen module. **F** The number of DE lncRNAs among different groups. The number of up-regulated and down-regulated DE lncRNAs was showed in bar plot.

**Additional file 3: Fig. S2.** CRLM1 regulates gene expression in HCT116 cells. **A**, **B** The top 10 representative KEGG terms of up- (**A**) and down-regulated genes (**B**) between CRLM1-OE and Ctrl. **C**, **D** The top 10 representative GO Biological Process terms of up- (**C**) and down-regulated genes (**D**) between CRLM1-OE and CRLM1-antisense-OE. **E**, **F** CRLM1-regulated genes mRNA expression was examined using qRT-PCR. **G**, **H** Gene expression level profile of genes involved in differentially expressed genes upon CRLM1-OE, metastasis-related genes from HCMDB database and CRLM1 co-expressed genes in 54 samples of GEO dataset (**G**) and CRLM1-OE RNA-seq dataset (**H**). **I** Box plot showing expression level of PPAP2B in Ctrl and CRLM1-OE samples.

**Additional file 4: Fig. S3.** CRLM1–chromatin interaction. **A** The Genome was divided for each 10 kb. Scatter plot showing the RPKM of each part between IP and Input samples. **B** The top 10 representative KEGG terms of all CRLM1-bound genes. **C** CRLM1-binding genes mRNA expression was examined using qRT-PCR. **D–F** Visualization of CRLM1 binding profiles on CRLM1 (**D**), AP4B1-AS1 (**E**), and ATP5F1 (**F**).

**Additional file 5: Fig. S4.** CRLM1 promotes hnRNPK promoter occupancy. **A** Venn diagram of hnRNPK binding genes in this work and the nearest genes to hnRNPK binding sites for quiescent cells or serum stimulated cells. **B** The top 10 representative GO Biological Process terms of all hnRNPK binding genes with CRLM1-OE. **C** Visualization of hnRNPK binding profiles on KLF6. **D** Visualization of hnRNPK binding profiles on ZFP36 and PLEKHG2.

**Additional file 6: Fig. S5.** CRLM1 and hnRNPK co-regulate gene expression in CRC cells. **A** Relative expression of HNRNPK in HCT116 cells after it was over-expressed was determined by RNA-seq data (left panel) and qRT-PCR (middle and right panel). **B** Heatmap shows the Person correlation between samples. CRLM1_hnRNPK_3 and Ctrl_hnRNPK_3 were not well correlated with their repetition and were not used in following analysis. **C**, **D** The top 10 representative GO Biological Process terms of up-regulated (**C**) and down-regulated (**D**) genes, comparing Ctrl_hnRNPK with Ctrl_NC samples. **E**, **F** The top 10 representative GO Biological Process terms of up-regulated (**E**) and down-regulated (**F**) genes, comparing CRLM1_hnRNPK with CRLM1_NC samples.

**Additional file 7: Table S1.** Probe sequences used to capture lncRNA-CRLM1 and LacZ in HCT116 cells. **Table S2.** Primer table that was used for qPCR experiment. **Table S3.** Primer table that was used for hnRNPK-RIP-qRT-PCR experiment. **Table S4.** 309 lncRNAs in MEgreen module related to metastasis using WGCNA. **Table S5.** DE lncRNAs between metastasis and primary tumor samples. **Table S6.** DEGs between CRLM1-OE and Ctrl samples. **Table S7.** CRLM1-bound peaks from ChIRP-seq data. **Table S8.** CRLM1-interacted proteins using mass spectrometry (ChIRP-MS). **Table S9.** HNRNPK_Ctrl-bound peaks from ChIP-seq data. **Table S10.** HNRNPK_CRLM1(OE)-bound peaks from ChIP-seq data. **Table S11.** DEGs between CRLM1-NC and Ctrl_NC samples. **Table S12.** DEGs between Ctrl_hnRNPK and Ctrl_NC samples. **Table S13.** DEGs between CRLM1_hnRNPK and CRLM1_NC samples. **Table S14.** CRLM1-HNRNPK up-regulated genes. **Table S15.** CRLM1-HNRNPK down-regulated genes.

## Data Availability

The sequenced RNA-seq, ChIP-seq, and ChIRP-seq data in this study have been deposited on NCBI Gene Expression Omnibus (GEO) database with an access number GSE167224. The data which support the findings of this study are available from the corresponding author on reasonable request.

## Abbreviations

CRLM: Colorectal liver metastases
lncRNAs: Long non-coding RNAs
CRC: Colorectal cancer
NC: Negative control
RNA-seq: RNA sequencing
ChIRP: Chromatin isolation by RNA purification
qRT-PCR: Quantitative real-time PCR
EMT: Mesenchymal transition
miRNAs: MicroRNAs
STR: Short tandem repeat
CBTCCCAS: Cell Bank, Type Culture Collection, and Chinese Academy of Sciences
siRNAs: Short interference RNAs
PI: Propidium iodide
FISH: Fluorescence in situ hybridization
IF: Immunofluorescence
ChIP: Chromatin immunoprecipitation
DEGs: Differentially expressed genes
RIP: RNA immunoprecipitation
PCA: Principal component analysis
TPM: Tags per Million
GSEA: Gene set enrichment analysis
TCGA: The Cancer Genome Atlas
WGCNA: Weighted gene co-expression network analysis
PPAP2B/PLPP3: Gene phospholipid phosphatase 3
KEGG: Kyoto Encyclopedia of Genes and Genomes
GO: Gene ontology
BP: Biological process
HCMDB: Human cancer metastasis database
MACS2: Model-based Analysis of ChIP-Seq 2
ChIRP-MS: ChIRP and mass spectrometry
RBPs: RNA binding proteins
mCRC: Metastatic colorectal cancer
