# Supplementary material for: LncRNA CRLM1 inhibits apoptosis and promotes metastasis through transcriptional regulation cooperated with hnRNPK in colorectal cancer
Source: Cell Biosci. 2022 Jul 30;12:120. doi: 10.1186/s13578-022-00849-9 (PMC9338583; doi:10.1186/s13578-022-00849-9)
Supplement: Supplementary file 1 — Additional file 1. Supplementary methods. [file 13578_2022_849_MOESM1_ESM.docx]

**Supplementary methods**

**Retrieval and process of public data**

GEO RNA-seq dataset (GSE50760) including 54 samples (18 normal colon, 18 primary tumor, and 18 liver metastasis tissue) across 18 colorectal cancer patients were used for lncRNA identification. Public sequence data files were downloaded from the Sequence Read Archive (SRA). SRA Run files were converted to fastq format with NCBI SRA Tool fastq-dump (https://ncbi.github.io/sra-tools/). The raw reads were trimmed of low-quality bases using a FASTX-Toolkit (v.0.0.13; <http://hannonlab.cshl.edu/fastx_toolkit/).> Then the clean reads were evaluated using FastQC (http://www.bioinformatics.babraham.ac.uk/projects/fastqc).

**Reads alignment and differentially expressed gene (DEG) analysis**

For GEO dataset, clean reads were aligned to the human GRch38 genome with Gencode annotation (v23) by TopHat2 [1] allowing 4 mismatches. Uniquely mapped reads were ultimately used to calculate read number and reads per kilobase of exon per million fragments mapped (RPKM) for each gene. The expression levels of genes were evaluated using RPKM. The software edgeR [2], which is specifically used to analyze the differential expression of genes, was applied to screen the RNA-seq data for DEGs. The results were analyzed based on the fold change (FC≥2 or ≤0.5) and *p*-value≤0.01 to determine whether a gene was differentially expressed.

**LncRNA prediction and direction identification**

To systematically analyze the lncRNA expression pattern, we used a pipeline for lncRNAs identification similar as previously reported [3], which was constructed based on the cufflinks software [4]. All steps of the pipeline have been shown in Figure S1A.

**WGCNA and co-expression analysis**

To fully understand the gene expression pattern, we applied weighted gene co-expression network analysis (WGCNA) [5] to cluster genes that have similar expression pattern with default parameters. All expressed genes were used as input data. Eigengenes for each clustering module was used as the representative expression pattern of genes in each module. To explore the co-expressed genes of lncRNAs, we calculated the Pearson’s correlation coefficients (PCCs) between them. Co-expressed gene pairs were screened out with the criteria: |Pearson’s correlation value| >=0.7 and pvalue <= 0.01.

**Analysis of RNA-seq data produced in this study**

Raw reads containing more than 2-N bases were first discarded. Adaptors and low-quality bases were then trimmed from the raw sequencing reads using the FASTX-Toolkit. Short reads less than 16 nt were also dropped. Clean reads were aligned and quantified as same as GEO dataset. For DEG analysis, the fold change (FC≥2 or ≤0.5) and false discovery rate (FDR≤0.05) was used to determine whether a gene was differentially expressed.

**Functional enrichment analysis**

To sort out functional categories of DEGs, Gene Ontology (GO) terms and KEGG pathways were identified using KOBAS 2.0 server [6]. Hypergeometric test and Benjamini-Hochberg FDR controlling procedure were used to define the enrichment of each term. Reactome (http://reactome.org) pathway profiling was also used for functional enrichment analysis of the sets of selected genes.

**Analysis of CRLM1 ChIRP-seq data**

Adaptors and low-quality bases were trimmed from raw sequencing reads using cutadapt [7] (version 1.8.1). Reads were aligned to the human GRCh38 genome using Bowtie2 [8]. Then SAM files of mapping results were generated for peak calling. Peaks of CRLM1_IP and LacZ group (each group contains two repetitions) were called using MACS2 (version 2.2.5) [9] against its corresponding Input with QVALUE cutoff 0.01. CRLM1_IP specific peaks not overlapped with LacZ peaks were used for further analysis. Peaks located on gene body or 5k of upstream of TSS was annotated with the gene.

**Analysis of HNRPK ChIP-seq data**

Adaptors and low-quality bases were trimmed from raw sequencing reads using cutadapt (version 1.8.1). Reads were aligned to the human GRCh38 genome using Bowtie2. Then SAM files of mapping results were generated for peak calling. Peaks of Ctrl_IP, CRLM1_IP and Anti_IP group (each group contains two repetitions) were called using MACS2 (version 2.2.5) against its corresponding Input with QVALUE cutoff 0.01. Peaks located on gene body or 5k of upstream of TSS was annotated with the gene.

**References**

1. Kim D, Pertea G, Trapnell C, Pimentel H, Kelley R, Salzberg SL. TopHat2: accurate alignment of transcriptomes in the presence of insertions, deletions and gene fusions. Genome Biology. 2013; 14 (4): R36.

2. Robinson MD, McCarthy DJ, Smyth GK. edgeR: a Bioconductor package for differential expression analysis of digital gene expression data. Bioinformatics. 2010; 26 (1): 139-140.

3. Liu S, Wang Z, Chen D, Zhang B, Tian R, Wu J, et al. Annotation and cluster analysis of spatiotemporal- and sex-related lncRNA expression in Rhesus macaque brain. Genome Res. 2017; 27 (9): 1608-1620.

4. Trapnell C, Roberts A, Goff L, Pertea G, Kim D, Kelley DR, et al. Differential gene and transcript expression analysis of RNA-seq experiments with TopHat and Cufflinks. Nature protocols. 2012; 7 (3): 562-578.

5. Langfelder P, Horvath S. WGCNA: an R package for weighted correlation network analysis. BMC bioinformatics. 2008; 9: 559.

6. Xie C, Mao X, Huang J, Ding Y, Wu J, Dong S, et al. KOBAS 2.0: a web server for annotation and identification of enriched pathways and diseases. Nucleic Acids Research. 2011; 39 (Web Server issue): 316-322.

7. Martin M. Cutadapt removes adapter sequences from high-throughput sequencing reads. EMBnet journal. 2011; 17 (1): 10-12.

8. Langmead B, Salzberg SL. Fast gapped-read alignment with Bowtie 2. Nature methods. 2012; 9 (4): 357.

9. Zhang Y, Liu T, Meyer CA, Eeckhoute J, Johnson DS, Bernstein BE, et al. Model-based analysis of ChIP-Seq (MACS). Genome biology. 2008; 9 (9): 1-9.
