# Supplementary figures and images for: LncRNA CRLM1 inhibits apoptosis and promotes metastasis through transcriptional regulation cooperated with hnRNPK in colorectal cancer
Source: Cell Biosci. 2022 Jul 30;12:120. doi: 10.1186/s13578-022-00849-9 (PMC9338583; doi:10.1186/s13578-022-00849-9)

**A**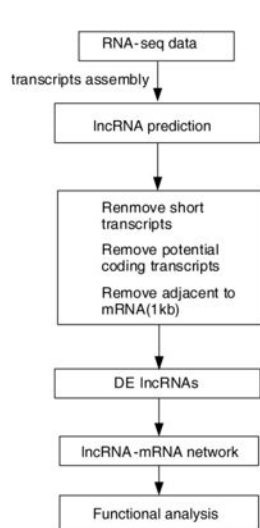**B**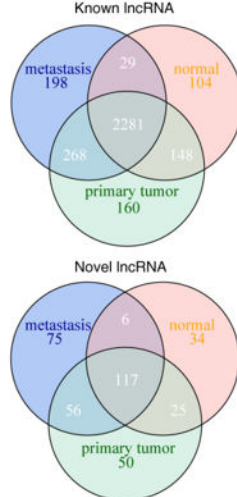**C**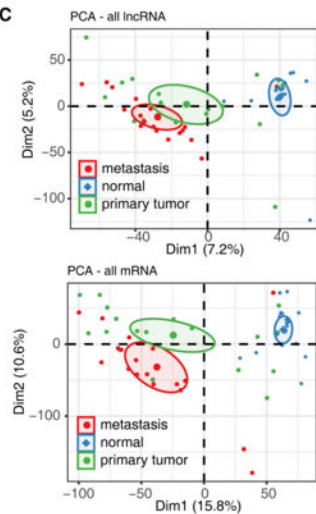**D**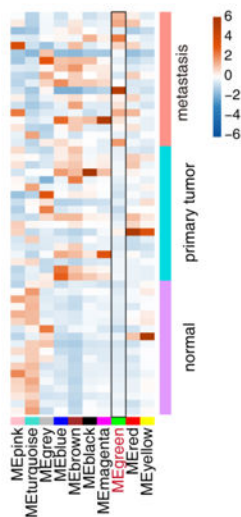**E**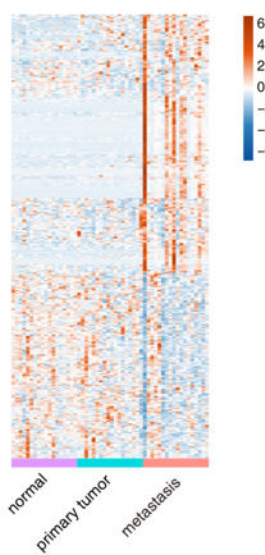**F**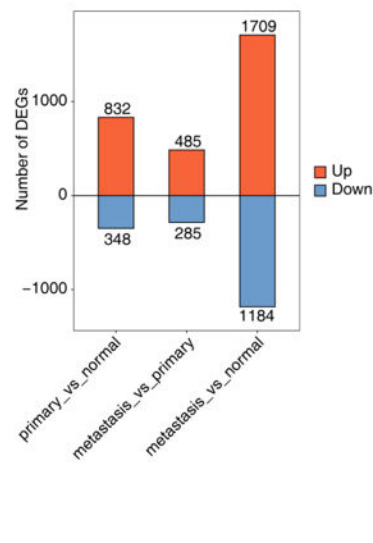

Supplement: Supplementary file 2 — Additional file 2: Fig. S1. CRLM1 is overexpressed in colorectal liver metastases and associated with poor survival. A Illustration of bioinformatics analysis pipeline for the identification and functional annotation of lncRNA genes expressed in CRC samples. B Venn diagram of detected known lncRNA (left) and novel lncRNA (right) in normal, primary tumor and metastasis samples. At least two samples with RPKM ≥ 0.2 was considered to be detected in the group. C Principal component analysis (PCA) of samples based on all mRNAs (up) and lncRNAs (down). The samples were grouped by disease state and the ellipse for each group is the confidence ellipse. D A Heat map of module Eigengenes sorted by average linkage hierarchical clustering. E The unsupervised hierarchical clustering heatmap of 54 samples based on genes involved in MEgreen module. F The number of DE lncRNAs among different groups. The number of up-regulated and down-regulated DE lncRNAs was showed in bar plot. [file 13578_2022_849_MOESM2_ESM.pdf]

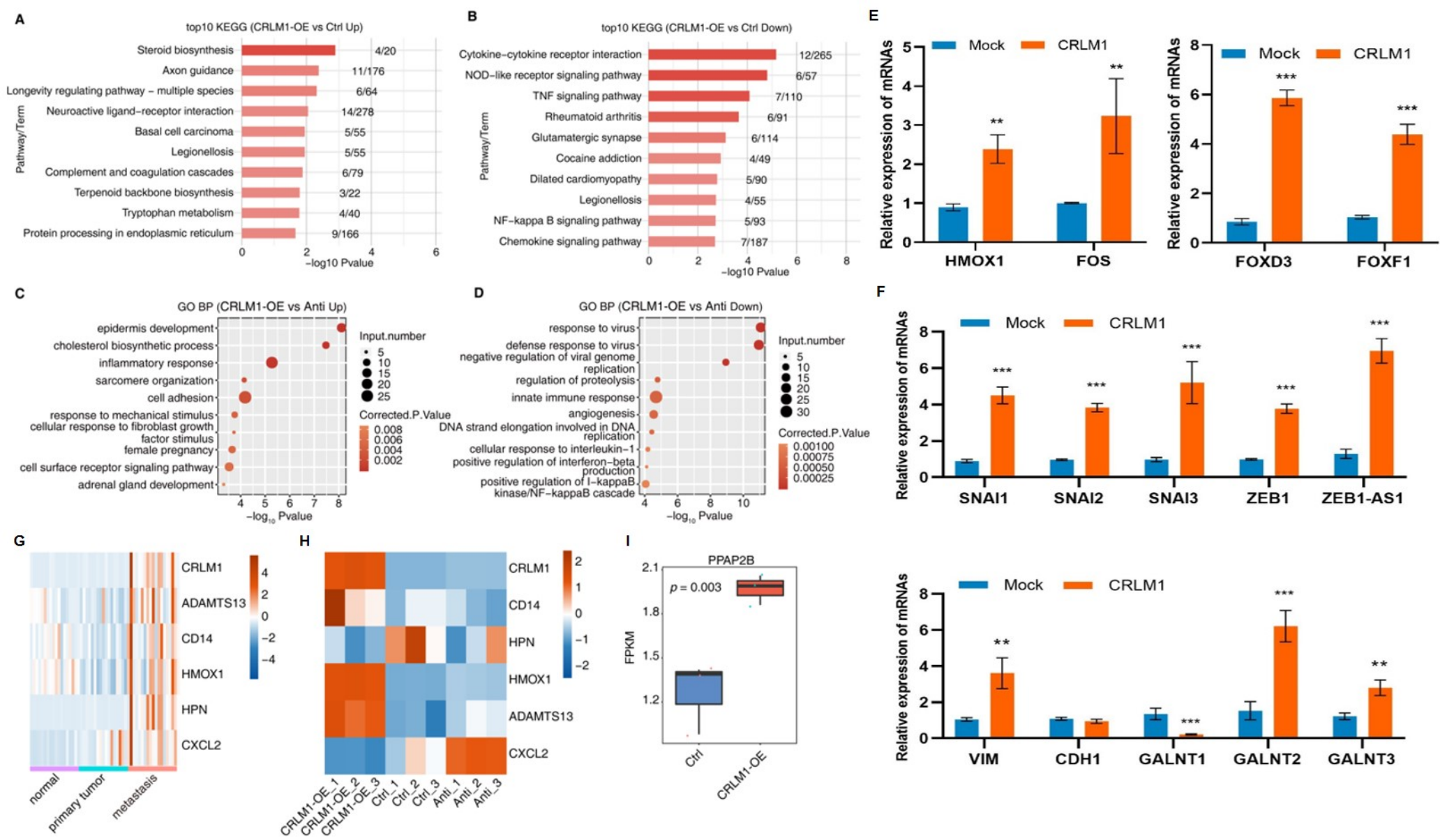

Supplement: Supplementary file 3 — Additional file 3: Fig. S2. CRLM1 regulates gene expression in HCT116 cells. A, B The top 10 representative KEGG terms of up- (A) and down-regulated genes (B) between CRLM1-OE and Ctrl. C, D The top 10 representative GO Biological Process terms of up- (C) and down-regulated genes (D) between CRLM1-OE and CRLM1-antisense-OE. E, F CRLM1-regulated genes mRNA expression was examined using qRT-PCR. G, H Gene expression level profile of genes involved in differentially expressed genes upon CRLM1-OE, metastasis-related genes from HCMDB database and CRLM1 co-expressed genes in 54 samples of GEO dataset (G) and CRLM1-OE RNA-seq dataset (H). I Box plot showing expression level of PPAP2B in Ctrl and CRLM1-OE samples. [file 13578_2022_849_MOESM3_ESM.pdf]

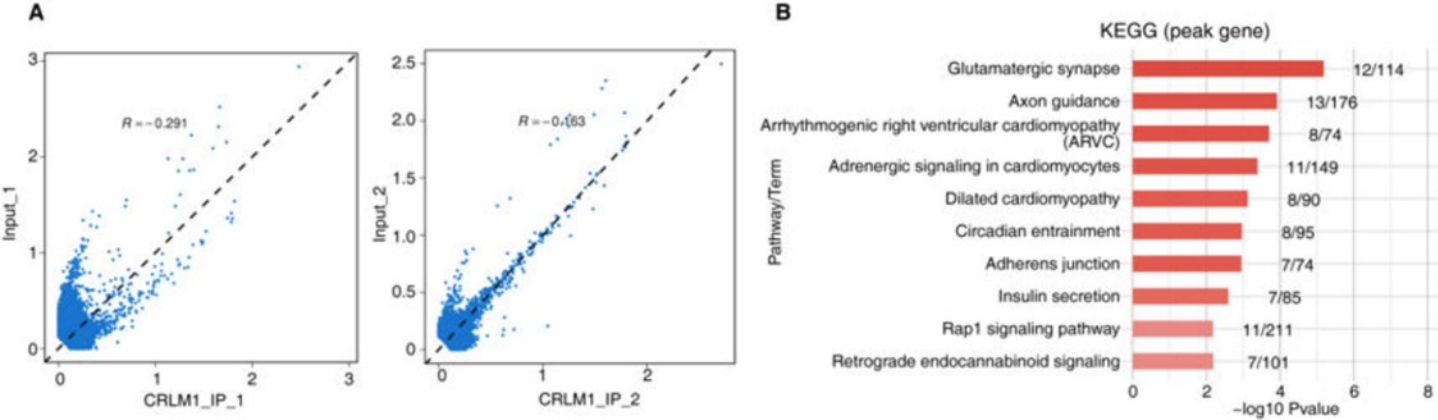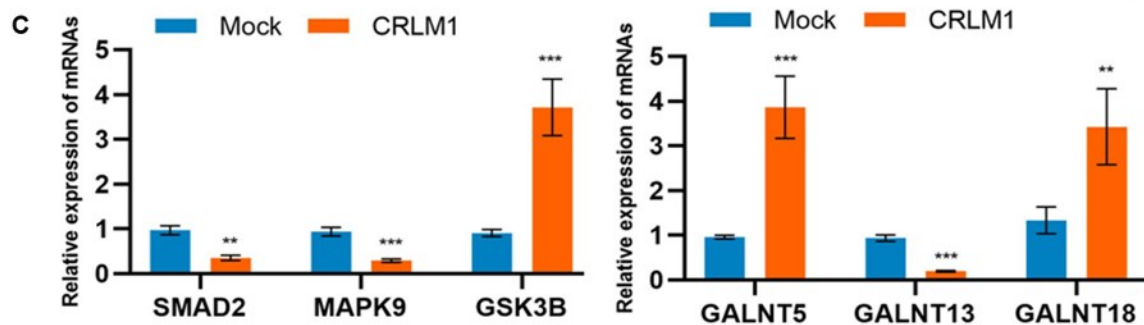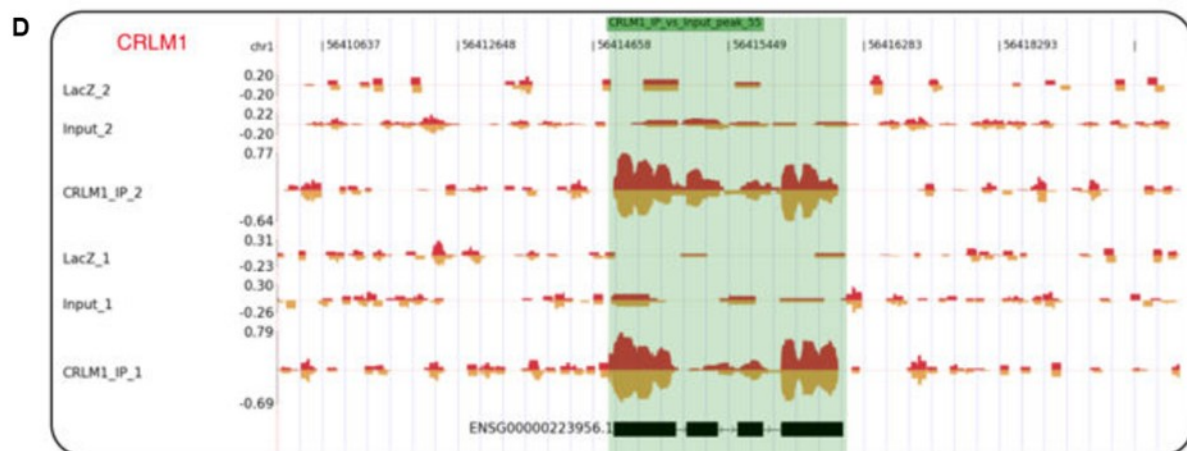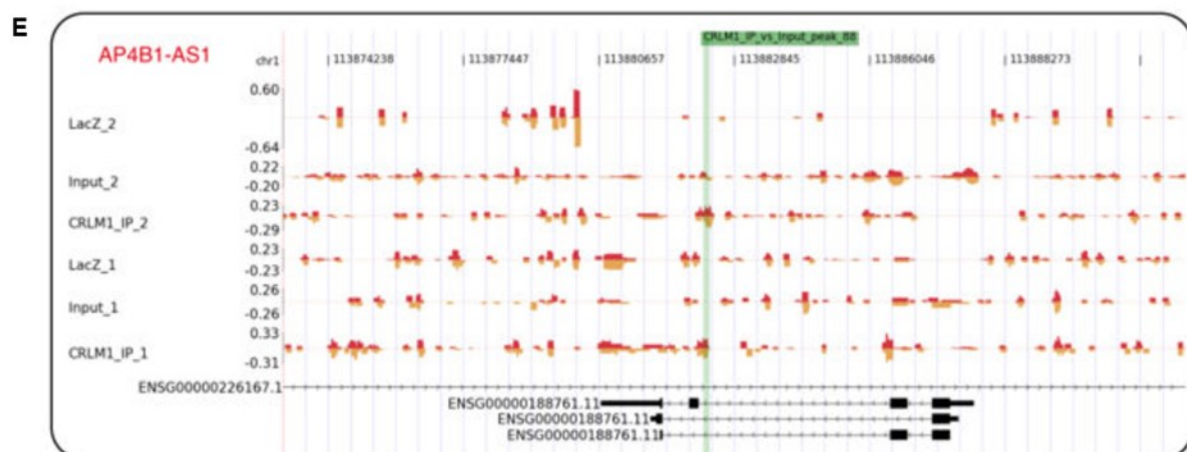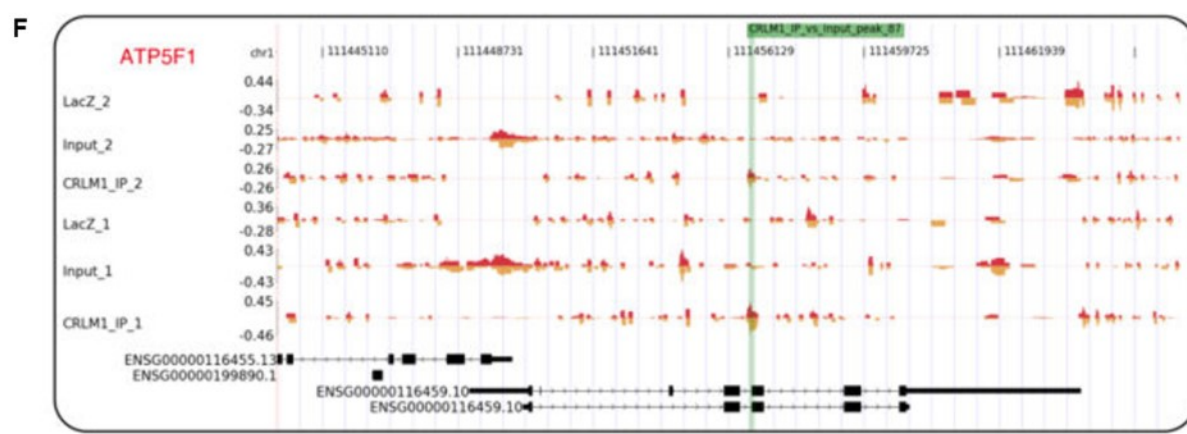

Supplement: Supplementary file 4 — Additional file 4: Fig. S3. CRLM1–chromatin interaction. A The Genome was divided for each 10 kb. Scatter plot showing the RPKM of each part between IP and Input samples. B The top 10 representative KEGG terms of all CRLM1-bound genes. C CRLM1-binding genes mRNA expression was examined using qRT-PCR. D–F Visualization of CRLM1 binding profiles on CRLM1 (D), AP4B1-AS1 (E), and ATP5F1 (F). [file 13578_2022_849_MOESM4_ESM.pdf]

**A**

Mikula, Michal, et al. (2013)

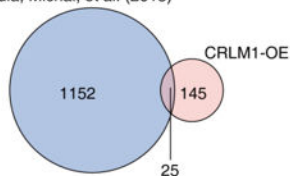**B**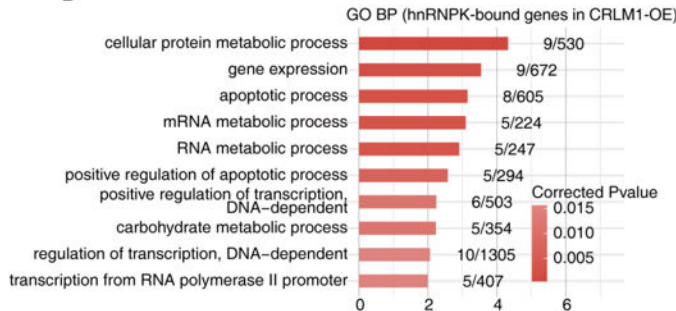**C**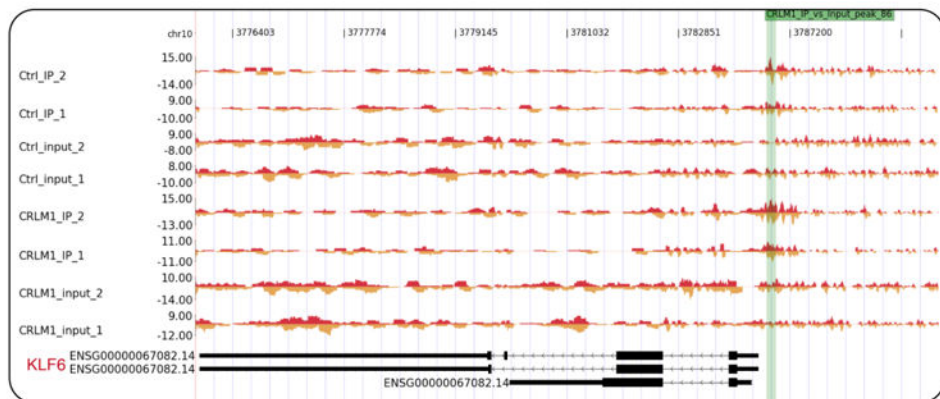**D**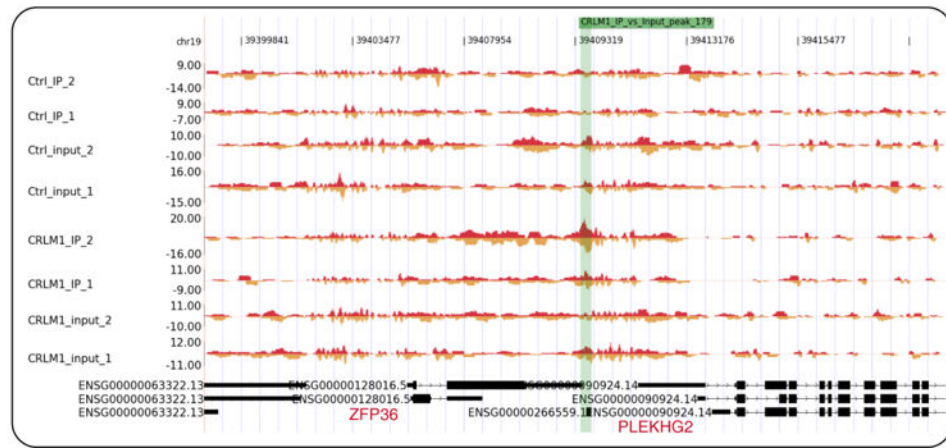

Supplement: Supplementary file 5 — Additional file 5: Fig. S4. CRLM1 promotes hnRNPK promoter occupancy. A Venn diagram of hnRNPK binding genes in this work and the nearest genes to hnRNPK binding sites for quiescent cells or serum stimulated cells. B The top 10 representative GO Biological Process terms of all hnRNPK binding genes with CRLM1-OE. C Visualization of hnRNPK binding profiles on KLF6. D Visualization of hnRNPK binding profiles on ZFP36 and PLEKHG2. [file 13578_2022_849_MOESM5_ESM.pdf]

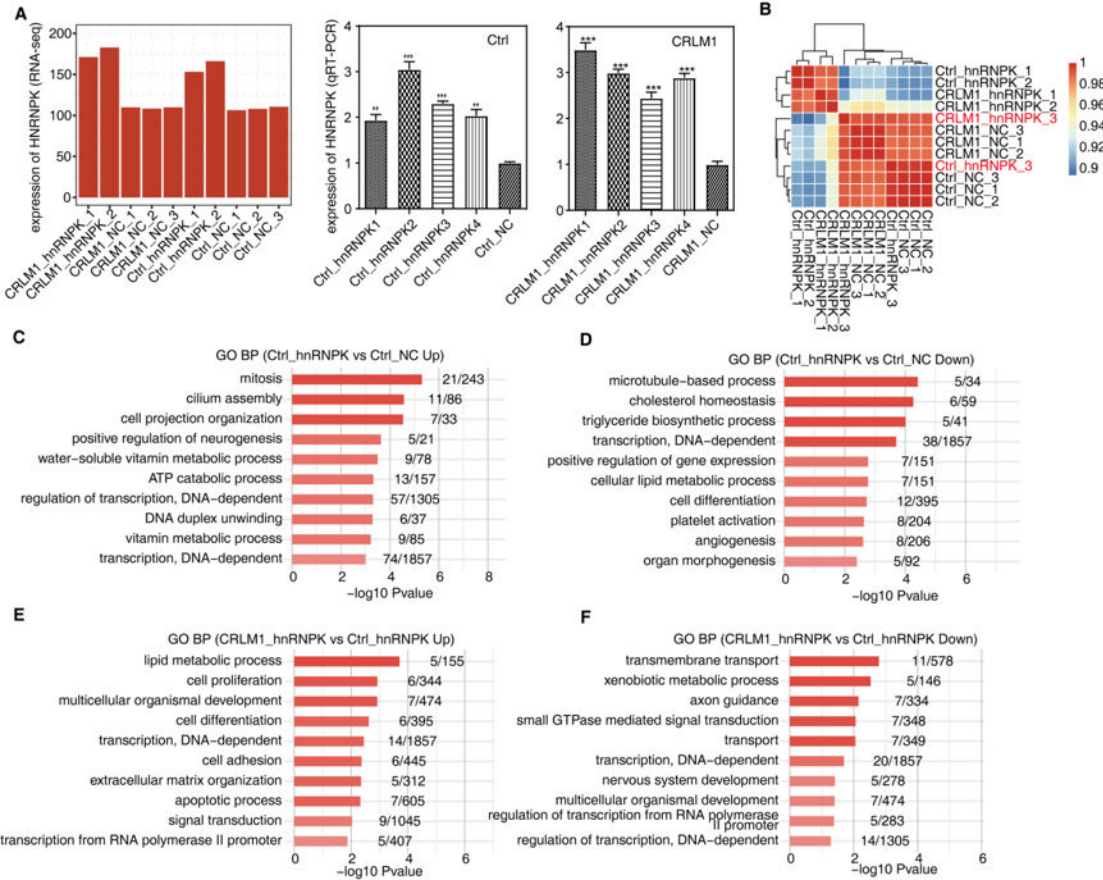

Supplement: Supplementary file 6 — Additional file 6: Fig. S5. CRLM1 and hnRNPK co-regulate gene expression in CRC cells. A Relative expression of HNRNPK in HCT116 cells after it was over-expressed was determined by RNA-seq data (left panel) and qRT-PCR (middle and right panel). B Heatmap shows the Person correlation between samples. CRLM1_hnRNPK_3 and Ctrl_hnRNPK_3 were not well correlated with their repetition and were not used in following analysis. C, D The top 10 representative GO Biological Process terms of up-regulated (C) and down-regulated (D) genes, comparing Ctrl_hnRNPK with Ctrl_NC samples. E, F The top 10 representative GO Biological Process terms of up-regulated (E) and down-regulated (F) genes, comparing CRLM1_hnRNPK with CRLM1_NC samples. [file 13578_2022_849_MOESM6_ESM.pdf]
